# Bioengineering commensal bacteria-derived outer membrane vesicles for delivery of biologics to the gastrointestinal and respiratory tract

**DOI:** 10.1080/20013078.2019.1632100

**Published:** 2019-06-24

**Authors:** Ana L. Carvalho, Sonia Fonseca, Ariadna Miquel-Clopés, Kathryn Cross, Khoon-S. Kok, Udo Wegmann, Katherine Gil-Cordoso, Eleanor G. Bentley, Sanaria H.M. Al Katy, Janine L. Coombes, Anja Kipar, Regis Stentz, James P. Stewart, Simon R. Carding

**Affiliations:** a Gut Microbes and Health Research Programme, Quadram Institute Bioscience, Norwich, UK; b Core Science Resources, Quadram Institute Bioscience, Norwich, UK; c Norwich Medical School, University of East Anglia, Norwich, UK; d Department of Biochemistry and Biotechnology, Universitat Rovira i Virgili, Tarragona, Spain; e Department of Infection Biology, University of Liverpool, Liverpool, UK; f Instiute of Veterinary Pathology, Vetsuisse Faculty, University of Zurich, Zurich, Switzerland

**Keywords:** Commensal bacteria, bacterial microvesicles, outer membrane vesicles, mucosal drug delivery, mucosal vaccines, therapeutic proteins

## Abstract

Gram-negative bacteria naturally produce and secrete nanosized outer membrane vesicles (OMVs). In the human gastrointestinal tract, OMVs produced by commensal Gram-negative bacteria can mediate interactions amongst host cells (including between epithelial cells and immune cells) and maintain microbial homeostasis. This OMV-mediated pathway for host-microbe interactions could be exploited to deliver biologically active proteins to the body. To test this we engineered the Gram-negative bacterium *Bacteroides thetaiotaomicron* (Bt), a prominent member of the intestinal microbiota of all animals, to incorporate bacteria-, virus- and human-derived proteins into its OMVs. We then used the engineered Bt OMVs to deliver these proteins to the respiratory and gastrointestinal (GI)-tract to protect against infection, tissue inflammation and injury. Our findings demonstrate the ability to express and package both *Salmonella enterica* ser. Typhimurium-derived vaccine antigens and influenza A virus (IAV)-derived vaccine antigens within or on the outer membrane of Bt OMVs. These antigens were in a form capable of eliciting antigen-specific immune and antibody responses in both mucosal tissues and systemically. Furthermore, immunisation with OMVs containing the core stalk region of the IAV H5 hemagglutinin from an H5N1 strain induced heterotypic protection in mice to a 10-fold lethal dose of an unrelated subtype (H1N1) of IAV. We also showed that OMVs could express the human therapeutic protein, keratinocyte growth factor-2 (KGF-2), in a stable form that, when delivered orally, reduced disease severity and promoted intestinal epithelial repair and recovery in animals administered colitis-inducing dextran sodium sulfate. Collectively, our data demonstrates the utility and effectiveness of using Bt OMVs as a mucosal biologics and drug delivery platform technology.

## Introduction

Described over 50 years ago by Bishop and Work (1965) as “extracellular glycolipids” produced by *Escherichia coli*, outer membrane vesicles (OMVs) are now considered to be naturally produced and secreted by most Gram-negative bacteria. Analyses of these 20–400 nm bilayered lipid membrane spherical structures have shown that they contain major components of the outer membrane such as lipopolysaccharide (LPS) in addition to the periplasmic contents of their ‘parent’ bacterium [1,2].

Historically, OMVs have been associated with pathogenesis and the storage and transportation of virulence factors produced by enteric Gram-negative pathogens including *Helicobacter pylori* (VacA), *Shigella dysenteriae* (Shiga toxin) and enterohemorrhagic *Escherichia. coli* (ClyA) [3–5]. Recently, this paradigm for OMV function has been questioned due to new evidence demonstrating a non-pathogenic, mutualistic role for the OMVs produced by commensal gut bacteria. Members of the genus *Bacteroides* exclusively package carbohydrate and protein hydrolases in OMVs that perform a ‘social function’ by providing substrates for utilization by other bacteria and contributing to microbiota homeostasis [6,7]. We [8,9] and others [10] have extended these observations providing evidence for a broader role of OMVs in gastrointestinal (GI)-tract homeostasis and the ability of *Bacteroides*-derived OMVs to influence host immune and epithelial cell responses.

OMVs can contain adhesins, sulfatases and proteases which facilitate their interaction with host epithelial cells, allowing them to enter these cells through numerous routes, including micropinocytosis, lipid raft- and clathrin-dependent endocytosis [11–13]. *Bacillus fragilis* OMVs containing polysaccharide A are detected by dendritic cells via Toll Like Receptor (TLR) 2 leading to enhanced T regulatory cell activity and production of anti-inflammatory cytokines (IL-10) that protect the host from experimental colitis [10]. We have demonstrated that OMVs produced by the human commensal bacterium *B. thetaiotaomicron* (Bt) can activate mammalian intestinal epithelial cell (IEC) intracellular Ca^2+^ signalling [8]. This host cell Ca^2+^ signalling response was dependent on Minpp, a novel constituent of these OMVs. Minpp is a homologue of a mammalian inositol phosphate polyphosphatase cell-signalling enzyme. Collectively, these findings demonstrate a non-pathogenic and beneficial role for OMVs produced by commensal *Bacteroides* species and are consistent with the concept that packaging of bioactive macromolecules in OMVs enables members of the intestinal microbiota to influence host cell physiology and establish bacteria-host mutualism [13].

It is feasible that this OMV-mediated pathway for host-microbe interaction could be exploited and used to deliver biologically active proteins to the body. Delivery to mucosal sites such as the GI- and respiratory tracts would be particularly valuable as they are vulnerable to injury and disease as a result of exposure to noxious environmental chemicals and pathogens [13,14]. Indeed, OMVs from *Neisseria meningitides* and *Vibrio cholera* have been incorporated into licensed vaccine formulations [15]; those derived from *N. meningitides* have been successfully used to immunise both children and adults and effectively control serogroup B meningococcal (MenB) disease outbreaks [16,17]. However, there are several limitations to using non-commensal, pathogen-derived OMVs as drug and vaccine delivery systems, particularly: their potential for unintended toxicity due to associated toxins; low expression levels of the heterologous antigens; variable efficacy depending on source and formulation; and the need for exogenous adjuvants in some applications. In principal, these limitations could be overcome by bioengineering the OMVs to improve their drug delivery capability [18]. Alternatively, non-pathogenic commensal bacteria could be used as a source of OMVs to reduce toxicity and improve safety.

To test this strategy we undertook a proof-of-principle study to determine the suitability of using OMVs produced by Bt to deliver bacteria-, virus- and human-derived proteins to the respiratory and GI-tract of mouse models (for respiratory influenza A virus infection and acute intestinal colitis) to protect them against infection, tissue inflammation and injury. Our findings, presented here provide evidence for the utility and effectiveness of using Bt OMVs as a mucosal biologics and drug delivery platform technology.

## Material and methods

### Bacteria strains, media and culture

Bt and its derivative strains (Table 1) were grown under anaerobic conditions at 37°C in an anaerobic cabinet. Bacterial starter-cultures were grown overnight in 20 ml “Brain Heart Infusion” (BHI) medium (Oxoid) supplemented with 15 µM haemin (Sigma-Aldrich) (BHIH). For OMV preparations, Bt cultures were inoculated with 0.5 ml of the starter-culture in a total volume of 500 ml BHI supplemented with 0.75 µM haemin. Cells were harvested after 16 h at an approximate OD_600_ _nm_ of 4.0, which corresponds to early stationary phase. Antibiotic-resistance markers in Bt were selected using erythromycin (5 μg/ml) and tetracycline (1μg/ml). *Escherichia coli* strains were grown in Luria-Bertani (LB) medium at 37°C with ampicillin 100 μg/ml (or 200 μg/ml trimethoprim for strain J53 [pR751]). *Lactococcus lactis* strain UKLc10 and its derivative strains were grown in M17 medium (Oxoid) supplemented with 5 g/l glucose at 30°C. Antibiotics were added as selection agents when appropriate: ampicillin 200μg/ml, erythromycin 5 μg/ml and chloramphenicol 10 μg/ml. The *E. coli* strain J53/R751 was supplemented with trimethoprim 200 μg/ml when grown for 18 h. The *E. coli* strain GC10 and the *L. lactis* strain UKLc10 were transformed by electroporation using a Gene Pulser II (Bio-Rad). For constructs relating to pUK200, the host *L. lactis* strain UKLc10 was used. Construction of other plasmids described below was achieved using *E. coli* strain GC10 as the host. Plasmids were mobilized from the *E. coli* into the Bt following a triparental filter mating protocol [19] using the helper strain J53/R751. All primers used are detailed in Table 2.

**Table 1.** Strains of bacteria used in this study.

Species	Strain	Plasmid	Protein expressed	Antibiotic selection*	Reference
*E. coli*	Rosetta 2(DE3) pLysS	pGH165	St OmpA	Amp, Cm	This study
	Rosetta 2(DE3) pLysS	pGH201	St SseB	Amp, Cm	This study
*Bt*	VPI-5482				DMSZ Collection
	GH290			Tet	This study
	GH490	pGH090		Ery	[21]
	GH484	pGH182	St OmpA	Ery	This study
	GH486	pGH183	St SseB	Ery	This study
	GH474	pGH173	Hu. KGF-2	Ery	This study
	GH503	pGH184	IAV H5F	Ery	This study
*S. enterica* ser. Typhimurim	SL1344				DMSZ collection
*L. lactis*	UKLc10				[22]

*Amp = ampicillin; Cm = chloramphenicol; Tet = tetracycline; Ery = erythromycin

**Table 2.** Primer sequences used in this study.

Primer	Sequence (5ʹ→ 3ʹ) ^a^
f-5ʹompA_SphI	ATCTGCATGCTTTCGAGGAAGAACCGATGGTTGC
r-5ʹompA_SalI	ATACGTCGACAATATAGCGGACTGCAATCC
f-3ʹompA_BamHI	ACTTGGATCCTTCTGAATCGTGTGGTATTGG
r-3ʹompA_SacI	ACTAGAGCTCATCTGTAGAGAAGAAACGGG
SPBTOmpA_fwd	CATGTTGCTGGCTTTTGCCGGCGTTGCGTCTGTCGCTTCTG CGCAGCAAACCGTGACTGTAACTGAATACGAGGTTATTCATATGTGACG
SPBTOmpA_rev	AATTCGTCACATATGAATAACCTCGTATTCAGTTACAGTCACGG TTTGCTGCGCAGAAGCGACAGACGCAACGCCGGCAAAAGCCAGCAA
OmpAST_fwd	TGACCATATGGCTCCGAAAGATAACACC
OmpAST_rev	GTCAGAATTCTTAAGCCTGCGGCTGAGTTA
SseB_fwd	TGACCATATGTCTTCAGGAAACATCTT
SseB_rev	TGACGAATTCATGAGTACGTTTTCTGCG
XhoI_STOmpA_rev	ATATCTCGAGGAAACTTAAGCCTGCGG
XhoI_SseB_rev	ATATCTCGAGATGAGTACGTTTTCTGCG

### Construction of a BT_3852 deletion mutant

A 1018 bp chromosomal DNA fragment upstream from *BT_3852* and including the first 18 nucleotides of its 5ʹ-end region was amplified by PCR using the primer pair f-5ʹompA_*Sp*HI, r-5ʹompA_*SalI*. This product was then cloned into the *Sp*HI*/SalI* sites of the *E. coli-Bacteroides* suicide shuttle vector pGH014 [20]. A 761 bp chromosomal DNA fragment downstream from *BT*_*3852*, including the last 46 nucleotides of the 3ʹ-end region, was amplified by PCR using the primer pair f-3ʹompA_*BamHI*, r-3ʹompA_*SacI* and was cloned into the *BamHI/SacI* sites of the pGH014-based plasmid. The resulting plasmid containing the Δ*BT*_*3852::tetQ* construct, was mobilized from *E. coli* strain GC10 into Bt by triparental filter mating [19], using *E. coli* HB101(pRK2013) as the helper strain. Transconjugants were selected on BHI-haemin agar containing gentamicin (200 mg/L) and tetracycline (1 mg/L). Determination of susceptibility to either tetracycline or erythromycin was done to identify recombinants that were tetracycline resistant and erythromycin susceptible after re-streaking transconjuguant bacteria on LB-agar containing tetracycline or both antibiotics. PCR analysis and sequencing were used to confirm allelic exchange. A transconjugant, GH290, containing the Δ*BT*_*3852::tetQ* construct inserted into the Bt chromosome was selected for further studies.

### Generation of recombinant BT strains

#### BT Salmonella OmpA/SseB

The *Bacteroides* expression vector pGH090 [21] was first digested with *NdeI* to remove this site by Klenow treatment and to create a blunt-ended fragment that was then religated. A sequence containing 90 bp of the *BT_3852* gene 5ʹ end (encoding a major outer membrane protein, OmpA) corresponding to the signal peptide sequence (SpOmpA) of the protein obtained from the microbial genome database (http://mbgd.genome.ad.jp/) was used to design the complementary oligonucleotide pair SPBTOmpA_fwd and SPBTOmpA_rev. Signal peptide prediction was obtained by SignalP (http://www.cbs.dtu.dk/services/SignalP/). After annealing of the oligonucleotides the resulting double-strand DNA contained *Eco*RI and *Sp*HI 5ʹ overhangs at each end. This linker was cloned into the *EcoRI/Sp*HI sites of the *Nde*I deleted version of pGH090, resulting in the pGH202 plasmid. The 1131 bp *Salmonella ompA* (without signal peptide) and the 591 bp *sseB* coding region were amplified by PCR from *S. enterica* ser. Typhimurium SL 1334 genomic DNA using the primer pairs OmpAST_fwd with OmpAST_rev, and SseB_fwd with SseB_rev, respectively. The resulting fragments were digested with *NdeI* and *EcoRI* and cloned into *Nde*I/*Eco*RI-digested pGH202, yielding plasmids pGH182 and pGH183, respectively. The latter plasmid was then transformed into *E. coli*-competent cells (GC10) by electroporation using a Gene Pulser II (Bio-Rad). Successful cloning was confirmed by sequencing. The plasmid was mobilized from *E. coli* to Bt using a triparental mating procedure [19], together with *E. coli* J53 (pR751); the correct structure of the Bt carrying pGH182 (GH484) was confirmed by sequencing.

#### BT IAV

A 635bp synthetic gene construct encoding a synthetic influenza (H5F; from IAV strain H5N1 [VN/04:A/VietNam/1203/04]) pre-fusion headless HA mini-stem N-terminally fused to the OmpA signal peptide of Bt was created *in silico* and its codon usage was optimised for expression in the same species. The resulting gene cassette was obtained by gene synthesis and subsequently cloned into the *E. coli* plasmid pEX-K168 (Eurofins). The cassette contained *Bsp*HI and *Eco*RI restriction sites at its 5ʹ and 3ʹ ends, respectively, allowing for the translational fusion of the gene to the start codon in the *Bacteroides* expression vector pGH090 [21]. The gene was excised from pEX-K168 using *Bsp*HI and *Eco*RI and ligated into the *Nco*I/*Eco*RI-restricted pGH090 expression vector, resulting in pGH184. Finally the sequence integrity of the cloned fragment was confirmed by sequencing. The plasmid was mobilized from *E. coli* into Bt through a triparental mating procedure.

#### BT KGF-2

A 581bp synthetic gene construct encoding the human fibroblast growth factor-10/keratinocyte growth factor-2 (KGF-2) N-terminally fused to the OmpA signal peptide of Bt was created *in silico* and its codon usage was optimised for expression in the same species. The resulting gene cassette was obtained by gene synthesis and subsequently cloned into the *E. coli* plasmid pEX-A2 (Eurofins) as described for the IAV constructs. The cassette contained *Bsp*HI and *Eco*RI restriction sites at its 5ʹ and 3ʹ ends, respectively, allowing for the translational fusion of the gene to the start codon in the *Bacteroides* expression vector pGH0902. The gene was excised from pEX-A2 using *Eco*53KI and *Eco*RI and ligated into pUK200 [22], which had been restricted with *Sma*I and *Eco*RI, resulting in plasmid pUK200_KGF-2. Next the KGF-2 cassette was excised from pUK200_KGF-2 through restriction with *Bsp*HI and *Eco*RI and subsequently ligated into the *Nco*I/*Eco*RI-restricted pGH090 expression vector, resulting in pGH173. Finally the sequence integrity of the cloned fragment was confirmed by sequencing. The plasmid was mobilized from *E. coli* into Bt using a triparental mating procedure.

### Expression and purification of recombinant StOmpA and StSseB

StOmpA was cloned into His6-tag expression vector pET-15b (Novagen). Briefly, PCR fragments incorporating the coding sequences of *ompA* and *sseB* genes were cloned into the NdeI/XhoI restriction sites of pET-15b and the resulting plasmids pGH165 and pGH201 transformed into Rosetta2 (DE3) pLysS cells (Table 1). Cultures of the resulting strains were induced at an of OD_600_ _nm_ of 0.6 by adding 1mM IPTG for 5 h after which time cells were harvested by centrifugation (5500 g for 20 min). The pellet was stored at −20°C for future use. StOmpA and StSseB proteins were purified under native conditions using protocols adapted from the QIAexpress Ni-NTA Fast Start Handbook (Qiagen) with the amount of protein recovered determined using the Bio-Rad Protein Assay.

### OMV isolation and characterisation

OMVs were isolated following a method adapted from Stentz et al. [20]. Briefly, cultures of Bt (500 mL) were centrifuged at 5500 g for 45 min at 4°C and the supernatants filtered through polyethersulfone (PES) membranes (0.22 μm pore-size) (Sartorius) to remove debris and cells. Supernatants were concentrated by ultrafiltration (100 kDa molecular weight cut-off, Vivaspin 50R, Sartorius), the retentate was rinsed once with 500 mL of PBS (pH 7.4) and concentrated to 1 mL (approx. 700 µg/ml total protein). The final OMV suspensions were filter sterilized (0.22 µm pore size). The protein content of the final OMV suspensions was determined using the Bio-Rad Protein Assay.

The distribution of heterologous proteins within Bt OMVs was established in a Proteinase K accessibility/protection assay [20]. Briefly, a suspension of 250 μg (total protein) of OMVs in 0.1 M phosphate/1 mM EDTA buffer (pH 7.0) was incubated for 1 h at 37°C in the presence of 100 mg/L proteinase K (Sigma-Aldrich). Proteinase K activity was stopped by addition of 1 mM phenylmethanesulfonyl fluoride (PMSF) and samples analysed by immunoblotting. The Sseb content of Bt OMVs was determined by targeted proteomics done by the Proteomics Facility, University Bristol, UK.

### Nanoparticle analysis

Size distribution of vesicles was performed on 1ml of OMV suspensions diluted 100 times with PBS. Videos were generated using a Nanosight nanoparticle instrument (NanoSight Ltd) to count the number of OMVs in each sample. A 1-min AVI file was recorded and analysed using NTA (Version 2.3 Build 0011 RC, Nanosight) software to calculate size distributions and vesicle concentrations using the following settings: calibration: 166 nm/pixel; blur: auto; detection threshold: 10, minimum track length: auto, temperature: 21.9C, viscosity: 0.96 cP. The accuracy of the measurement was confirmed using 100 nm silver nanoparticles (Sigma-Aldrich).

### Electron microscopy

The volume of OMV suspensions in PBS (1ml) was adjusted to 8.9 ml with PBS and then concentrated by ultracentrifugation; 150,000 g for 2 h at 4°C in a Ti70 rotor (Beckman Instruments). The vesicle containing pellet was resuspended in 200 µl of PBS. The OMV suspension was fixed for 1 h using 25% glutaraldehyde then centrifuged at 13,000g for 10 min. The OMV pellets were mixed 1:1 with molten 2% low gelling temperature agarose (TypeVII, Sigma), which was solidified by chilling and then cut into ~1 mm^3^ cubes. The sample pieces were then further fixed in 2.5 % glutaraldehyde in 0.1 M PIPES buffer for 16 h at 4°C after which time they were washed three times in 0.1 M PIPES buffer and dehydrated through a series of ethanol solutions (30, 50, 70, 80, 90%, and 3 times in 10 0%) after which the ethanol was replaced with a 1:1 mix of 100% ethanol:LR White medium grade resin and put on a rotator for 1 h. This was followed by a 1:2 and then a 1:3 mix of 100% ethanol:LR White resin mix and finally 100% resin, with at least 1 h between each change. The resin was changed twice more with fresh 100% resin with 8 h between changes. The sample pieces were each transferred into BEEM embedding capsules with fresh resin and polymerised for 16 h at 60°C. Sections of ~90 nm thick were cut using an ultramicrotome (Ultracut E, Reichert-Jung) with a glass knife and collected on film/carbon coated gold grids. A modified version of the Aurion Immunogold labelling (IGL) protocol (http://www.aurion.nl/the_aurion_method/Post_embedding_conv) was used with 1 h antibody incubations and detergent (0.1% TWEEN). The primary anti-Bt OmpA antisera was obtained by immunising rabbits with the peptide GGPREDGSYKQRWDYMN (Cambrige Research Biochemical), and was used at a dilution of 1/500. The secondary anti-rabbit Ig (GAR-10, Agar Scientific) was used at a dilution of 1/50. After antibody labelling, sections were stained with 2% uranyl acetate for 40 min and imaged in a FEI Tecnai G2 20 Twin transmission electron microscope at 200 kV.

### Immunoblotting

Bt cell and OMV extracts were obtained by sonication and the supernatants added to SDS Page loading buffer (NuPage) containing dithiothreitol (Invitrogen). Approximately 7 μg of the total protein was loaded onto 12% precast Tris-Glycine gels (Novex) and separated by electrophoresis at 180 volts for 40 min. The gel was then transferred onto a polyvinylidene difluoride (PVDF) membrane at 25 volts for 2 h in a solution containing Tris-Glycine Transfer Buffer (Novex). The membrane was blocked with 10% BSA in TBS-Tween (TBS [50 mM Tris-HCl; 150 mM NaCl; pH 7.5] with 0.05% Tween) by shaking for 30 min at 20°C. The blocking solution was then discarded and the membrane incubated for 16–18 h at 4°C in TBS-Tween with 5% BSA containing primary antibody (anti-*Salmonella* OmpA [Antibody Research Corporation], -KGF-2 [Peprotech] or -IAV or Anti-polyHisitdine Clone HIS-1 (Sigma-Aldrich). After washing with TBS-Tween, membranes were incubated in 5% BSA in TBS-Tween containing HRP-conjugated goat anti rabbit IgG (1:1000 dilution, ThermoFisher) for 1 h at 20°C. After 3 washes with TBS-Tween, SuperSignal West Pico chemiluminescent Substrate (ThermoFisher) was used to detect bound antibody.

### Mammalian cell culture

The human colonic epithelial cell line Caco-2 (ECACC 86,010,202) was cultured at 37°C and 5% CO_2_ in Dulbecco’s Modified Eagle Medium (DMEM) with 4.5 g/L glucose and L-glutamine (Lonza) supplemented with 5% foetal bovine serum (FBS, Lonza).

### Epithelial cell scratch assay

Caco-2 cells were grown in T25 flasks until they reached 90% confluency. Cells were digested using trypsin EDTA (200 mg/L, 170,000 U Trypsin/L, Lonza) and seeded onto 8-well µ-slides (Ibidi). Cells were grown until they formed a 90% confluent monolayer and then serum-starved for 8 h. A scratch was made on the monolayer using a sterile tip and cells were washed with PBS to remove cell debris. The remaining cells were incubated for 72 h in 1% FBS medium supplemented with heparin (300 µg/mL grade I-A, >180USP units/ml; Sigma-Aldrich) in the presence of PBS, naïve OMVs, KGF-2 OMVs or recombinant KGF-2 (500 ng/mL, PeproTech). Wound healing was monitored by taking images immediately after scratching (time 0 control) and every 24 hours using an Invertoskop ID03 inverted microscope (Carl Zeiss) and a Sony Xperia Z5 compact digital camera (Sony). The measurements of the recovered scratch area (pixel^2^) at each time point were analysed using ImageJ software. The experiment was performed in triplicate.

### Animal experiments

All animal experiments were done using 6 to 8 week old C57BL/6 male mice that were bred and maintained in animal facilities either at the University of East Anglia (UK) or the University of Liverpool. Mice were housed in individually ventilated cages and exposed to a 12 h light/dark cycle with free access to a standard laboratory chow diet. Animal experiments were conducted in full accordance with the Animal Scientific Procedures Act 1986 under UK Home Office (HMO) approval and HMO project license 70/8232 (UEA) and 70/8599 (UoL).

*OMV vaccines and vaccination*: To evaluate oral *Salmonella* OMV vaccine formulations, groups of mice (n = 5–6/group) were gavaged with either 100 μl containing approximately 70 µg of total protein and 10^10^ vesicles of StOmpA-OMVs or naïve OMVs in PBS. Prior to each immunization food was removed for approximately 4 h to decrease stomach acidity. Booster oral immunisations were given 1 and 2 months later. An additional control group of animals were immunised with StOmpA-OMVs via the intraperitoneal route. To assess intranasal immunisation with *Salmonella* and influenza virus OMV vaccine formulations, groups of mice (n = 5–10/group mice were anaesthetized then intranasally dosed with either StOmpA OMVs, StSseB OMVs, H5F OMVs, naïve OMVs (~70 µg of total protein) or PBS and 14 and 21 days later received booster immunizations. For infectious challenge with *Salmonella*, StOmpA-OMV orally or intraperitoneally (IP) immunised mice were orally administered 10^8^ CFU of *S. enterica* ser. Typhimurium SL1344 on day 70 and 5 days later the bacterial load in different tissues was determined. For infectious challenge with IAV, H5F-OMV immunised mice were anaesthetised on day 28 with ketamine via the intra-muscular route and inoculated intranasally with 10^3^ PFU A/PR/8/34 (PR8) H1N1 strain of IAV in 50 μl sterile PBS, which is equivalent to a 10-fold lethal dose. Weights of each animal were recorded from the day of challenge up until the end point at day 33 when the mice were euthanised. At necropsy, blood, serum and bronchoalveolar lavage fluid were taken for antibody and cytokine analyses and lung tissue was used to determine virus titre. For *in vivo* OMV trafficking studies, mice were intranasally administered with DiO-labelled H5F-OMVs and 1 and 5 days later OMV acquisition and uptake was determined using flow cytometry in: the macrophage and dendritic cells of the BAL; nasal associated lymphoid tissue (NALT); and cervical and mediastinal lymph nodes.

#### IAV quantification

Plaque assays were performed on homogenates of lung tissue from PR8-infected mice as described previously [23]. Briefly, viral samples from lungs were titrated in a 10-fold serial dilution from 10^1^ to 10^6^ in DMEM supplemented with TPCK-trypsin. Each dilution was incubated with MDCK cells in individual wells of a 24 well plate for 1 hour at 37**°**C, 5% CO_2_. The media was aspirated and replaced with overlay media containing 2.4% Avicel. Plates were incubated at 37**°**C, 5% CO_2_ for 72 hours. Avicel was aspirated, plates were washed and cells were fixed in acetone:methanol (60:40) for 10 min. Cells were allowed to air dry prior to staining with crystal violet for 10 minutes, washed and air dried. Plaques were counted and then multiplied by the dilution factor and the volume of virus plated to give viral titre (PFU/ml).

#### Acute colitis

The dextran sulphate sodium (DSS) induced mouse model of acute colitis was used to test the therapeutic potential of KGF-2-containing OMVs. Mice were divided into six groups (n = 5/grp) and administered with either PBS, naïve OMVs, KGF-2 OMVs, DSS + PBS, DSS + naïve OMVs or, DSS + KGF-2 OMVs for 7 days. Experimental colitis was induced in the appropriate treatment groups of mice by administration of 2.5% w/v DSS (36,000–50,000 Da, MP Biomedicals, USA) in drinking water *ad libitum* for 7 days. The other groups of mice received fresh water alone throughout the duration of the experiment. PBS and OMVs were administered by oral gavage (100 µL) on days 1, 3 and 5 and on day 7 mice were euthanized. Fresh faecal pellets were collected daily by placing individual mice in an empty cage without bedding material for 5–15 min. The extent of colitis was evaluated using a disease activity index (Table S1) comprising daily body weights, stool consistency and rectal bleeding assessments. At autopsy the colon was aseptically extracted and photographed, and the contents collected in sterile vials and stored at −80°C. The colon length was measured, and representative samples (0.5 cm length) were taken from the distal region for histology. Histological samples were fixed in 10% neutral buffered formalin and embedding in paraffin. Tissue sections (5 μm) were prepared from each block, stained with hematoxylin (Mayer’s hemalum, Merk) and eosin (Y-solution 0.5% aqueous, Merck) (H&E), and with Alcian blue (Sigma-Aldrich) and Nuclear Red (Sigma-Aldrich) to visualise goblet cells. Sections were observed under a DMI 3000B microscope at 40X magnification (Leica) and assessed in a blinded fashion. The histological changes were scored (Table S2) and goblet cells were enumerated using ImageJ software.

#### Antibody ELISA

ELISA plates were coated with target antigens (UV inactivated IAV [PR8] virus or H5 (H5N1) (A/Vietnam/1203/2004) Recombinant Protein (P5060, 2B Scientific Ltd), *Salmonella* OmpA or SseB proteins) in 0.1M NaHCO_3_ and incubated for 12–16 hours at 4**°**C. Plates were washed three times with PBS that had been supplemented with 0.05% Tween 20 (PT), and then incubated with blocking solution (PBS with 2% BSA) for 3 h at 20°C, and then washed six times with PT. Fecal pellets were homogenized in phosphate-buffered saline (pH, 7.2) with soybean trypsin inhibitor (0.5 mg/mL; Sigma), phenylmethylsulfonyl fluoride (0.25 mg/mL; Sigma), 0.05 M EDTA, and 0.05% Tween 20 (Sigma). The fecal homogenates and bronchoalveolar lavage (BAL) and serum samples were diluted in PBS with 1% BSA, 0.05% Tween (PBT) and added to the plate wells and incubated for 12–16 h at 4**°**C. Immune serum and BAL from PR8 IAV-infected mice were used as reference samples for analysing anti-IAV antibody responses in H5F-OMV-immunised animals. Plates were then washed six times with PT and incubated with PBT containing either HRP-anti-mouse IgG (1:1000, Thermo-Fisher) or HRP-anti-mouse IgA (1:1000, Life Technologies) for 20 min at 20°C. Plates were again washed six times with PT then incubated in darkness with TMB High Sensitivity substrate solution (BioLegend) for 30 min at 20°C. The reaction was stopped by the addition of 2 N H_2_SO_4_ and the optical density was measured at 450 nm using a TECAN infinite f50 spectrophotometer (Männedorf, Switzerland). Abcam’s IgA Mouse ELISA Kit was used to determine total IgA in salivary glands and BAL

#### Flow cytometry

Approximately 1 × 10^6^ tissue-derived cells were incubated in PBS supplemented with 2% FCS (PBS-FCS) for 15 min at 4**°**C prior to the addition of fluorochrome-conjugated monoclonal antibodies specific for CD11b (clone M1/70, eBioscience) CD11c (clone N418, eBioscience), or CD103 (clone 2E7, eBioscience) in PBS-FCS and incubated for 30 min at 4**°**C in darkness. Cells were then washed in PBS-FCS and fixed in PBS supplemented with 4% paraformaldehyde for 15 min at 20 °C prior to analysis on a MACSQuant Analyzer 10 (Miltenyi Biotech). Data were analysed using FlowJo.

### Immunohistology

From all mice, the entire, skinned heads were fixed in 10% buffered formalin for 48 h. Subsequently, approximately 2 mm slices were prepared by sagittal sections, using a diamond saw (Exakt Band System 300 CL; EXAKT Technologies Inc.), yielding a total of six sections from the tip of the nose to the foramen occipitale magnum. Sections were gently decalcified for 7 days in RDF Mild Decalcifier (CellPath Ltd) at room temperature. Likewise, thoracic organs (lungs, lymph nodes, heart and thymus) were removed en bloc, fixed for 24 h in 10% buffered formalin and trimmed. Head and organ specimens were then routinely paraffin wax embedded. Consecutive sections (3–5 µm) were prepared and were stained with haematoxylin eosin for histological examination, or subjected to immunohistological staining. Immunohistology (IH) was performed using the horseradish peroxidase method as previously described [24,25]. Primary antibodies used were rat anti-mouse CD45R (clone B220, BD Biosciences; B cells), rabbit anti-CD3 (clone SP7; Bioscience; T cells) and rabbit anti-Iba-1 (Wako; macrophages and dendritic cells).

### Statistical analysis

Data were subjected to the D’Agostino & Pearson omnibus normality test. One-way ANOVA followed by a Tukey’s multiple comparison post hoc tests were made using GraphPad Prism 5 software. Statistically significant differences between two mean values were established by a p-value < 0.05. Data are presented as the mean ± standard deviation.

## Results

### Characteristics and physical properties of BT OMVs

**Figure 1.**
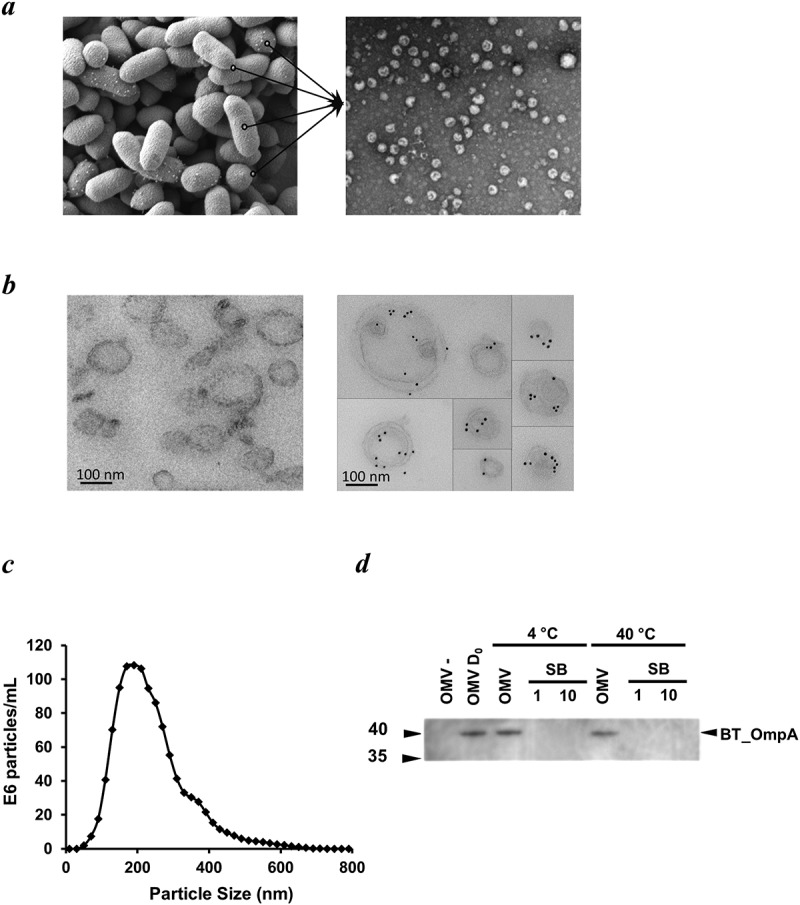
Appearance, size, structure and stability of Bt OMVs. (a) Electron microscopy (EM) of Bt cells showing vesicles budding from their surface before release into the milieu (lines in left panel), and EM image of OMVs extracted from cell culture supernatants (right panel). (b) Immunodetection of naïve Bt OMVs using colloidal gold anti-rabbit Ig to detect binding of rabbit anti-Bt OmpA antisera (right panel). Left panel shows absence of staining of OMVs produced by an OmpA deletion mutant of Bt. (c) Size distribution of OMVs produced by *Bt* determined by nanoparticle tracking analysis. (d) Thermostability of OMVs at day 0 (OMV D_0_) and after storage of OMV suspensions at 4°C or 40°C for 30 days as measured using immunoblotting to detect OmpA in extracts of naïve OMVs (OMV) or OMVs of *ompA* deletion mutants (OMV-), and of neat (1) or ten-times concentrated (10) OMV storage buffer (SB) (PBS was the storage buffer).

Using electron microscopy we showed that OMVs budded off from the outer membrane of Bt during its growth cycle (Figure 1(a)) and could be recovered by filtration and ultracentrifugation from early stationary growth phase cultures (see Material and Methods). Bt OMVs were identified by their characteristic double membrane of their parental cells and by immunoreactivity with anti-Bt OmpA antisera (Figure 1(b)). Bt OMVs ranged in size from approximately 100 nm to greater than 400 nm with a mean size of 237 nm (Figure 1(c)). Bt OMVs are also highly stable with minimal loss of luminal proteins (<10% of total protein or heterologous protein) detected after exposure to an elevated ambient temperature of 40 °C for up 30 days (Figure 1(d)).

### Heterologous bacterial, viral and human proteins expressed in BT and incorporated into OMVs

To test whether the delivery of a wide range of heterologous antigens into Bt OMVs was possible we compared delivery of a number of selected candidate vaccine antigens. These included antigens for the important bacterial and viral pathogens *S. enterica* ser. Typhimurium *enterica* and IAV, respectively. For a human protein we chose keratinocyte growth factor-2 (KGF-2). For *S. enterica* ser. Typhimurium we chose the outer membrane protein, OmpA and the SPI-2 translocon subunit (SseB) because they elicit antibody and T cell responses and confer some degree of protective immunity in mice, and because antibody responses in humans correlate with immune protection [26–34]. For IAV, the H-stalk protein H5 of the H5N1 VN/04:A/VietNam/1203/04 subtype was selected as it confers robust protection against challenge by multiple strains of IAV and can reduce lung viral titres by 3-fold [35–37]. We also chose KGF-2 because it is essential for epithelial cell proliferation and preserving the integrity of the intestinal mucosa [37], and has therapeutic potential for the treatment of inflammatory bowel disease [38].

**Figure 2.**
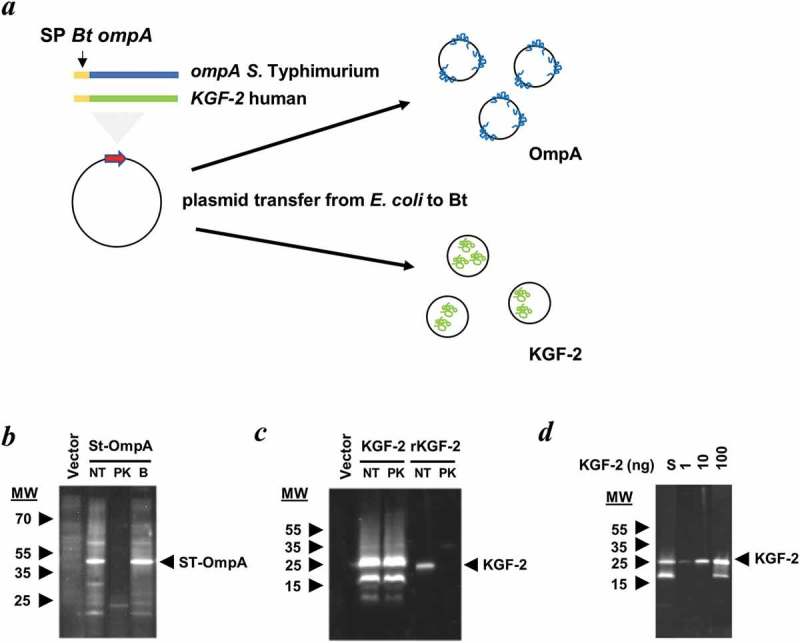
Expression of heterologous proteins in Bt OMVs. (a) Schematic of cloning procedure for the export of proteins of interest into the lumen or at the surface membrane of OMVs. The secretion peptide of Bt OmpA (SP BtompA) is indicated in yellow and fused at the N-terminus of the gene of interest. (b and c) Determination of protein location after treatment with proteinase K (PK). Immunoblotting of StOmpA (b) and KGF-2 (c) with and without pre-treatment of OMV suspensions with proteinase K. NT: not treated; PK: + Proteinase K; B: PK buffer alone. (d) KGF-2 quantification within OMVs. Comparison of recombinant KGF-2 (1–100 ng) with 10 µl of 1 ml OMV suspension (S).

Mini-genes encoding the selected bacterial, viral and human proteins were cloned downstream of sequences encoding the N-terminal signal peptide of the major outer membrane protein OmpA (BT_3852), the products of which are contained within the lumen or outer membrane of OMVs (Figure 2(a)). These constructs were created in *L. lactis* (for KGF-2) or *E. coli* (for OmpA, SseB and HF) hosts and then mobilised into Bt via a triple filter mating protocol using a helper strain. Immunoblotting of whole cell and OMV lysates of recombinant Bt strains confirmed expression of OmpA (Figure 2(b)) and KGF-2 proteins (Figure 2(c)). The luminal versus outer membrane distribution of these heterologous proteins in Bt OMVs was established using a protease protection assay which showed that *Salmonella* OmpA distribution was associated with the outer membrane (Figure 2(b)) whereas KGF-2 (Figure 2(c)) was contained within the lumen of OMVs. SseB expression was undetectable in OMV preparations using immunoblotting, but was detectable by liquid chromatography and mass spectrometry- (LC-MS) based proteomics, which also established its localization to the lumen of OMVs (Supplementary Table S3).

### BT OMVs have inherent adjuvanticity

**Figure 3.**
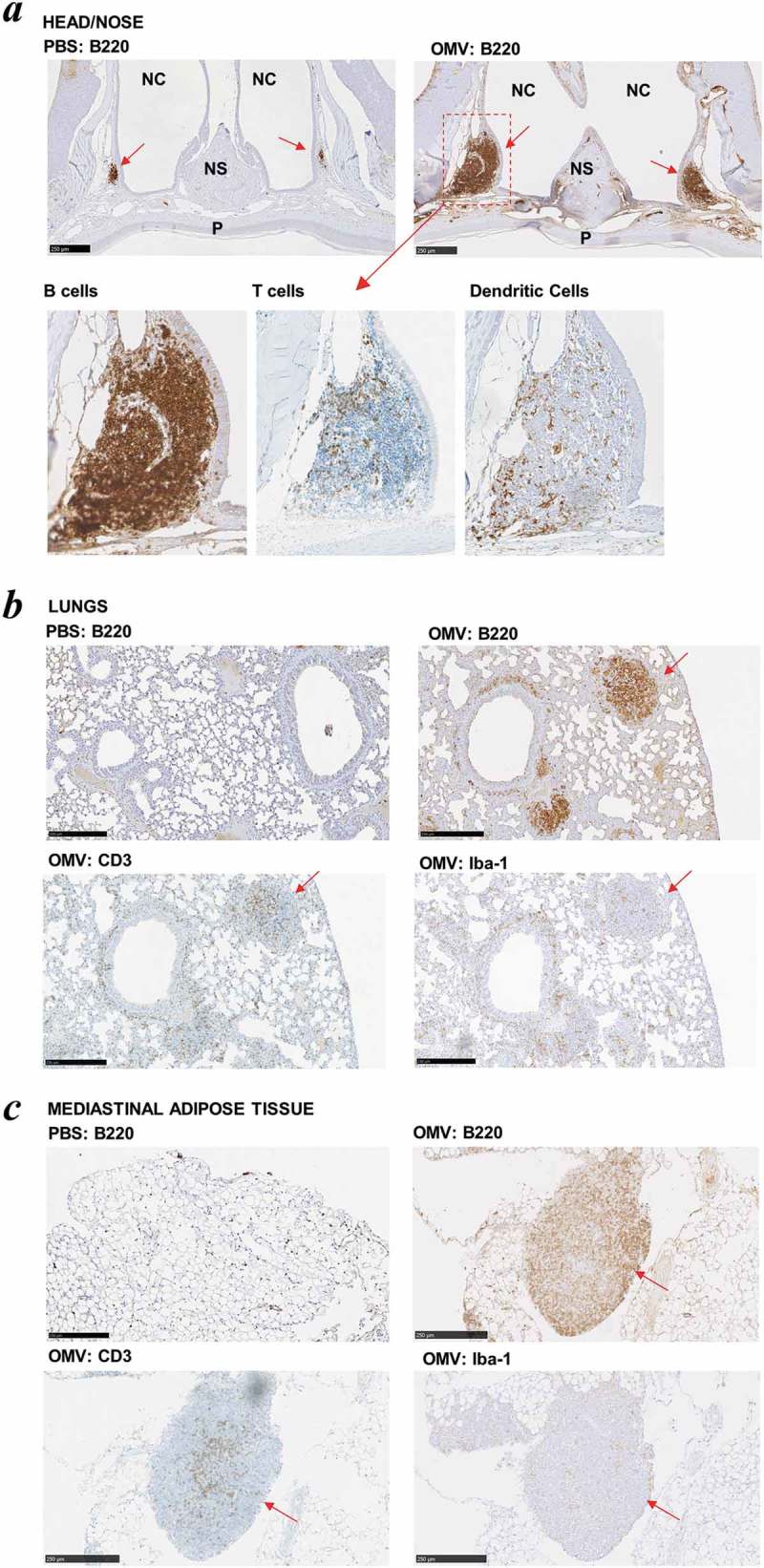
Intrinsic adjuvanticity of Bt OMVs. (a) Mice (n = 5) were intranasally adminstered PBS alone native Bt OMVs (OMV) in PBS and 5 days later heads and thoracic tissue was processed for immunohistology to visualise immune cell activation and formation of organised lymphoid tissue containing CD45R^+^ B cells (B220), CD3^+^ T cells (CD3) and macrophages/dendritic cells (Iba-1) in the nasal associated lymphoid tissue (a) the lung parenchyma (b) and mediastinal adipose tissues (c). Red arrows define nasal-associated lympoid tissue (NALT), bronchus-associated lymphoid tissue (BALT) and fat-associated lymphoid tissue (FALC) in a, b and c respectively. NC: nasal cavity, NS: nasal septum, P: hard palate.

Many conventional vaccines rely on the inclusion of adjuvants to enhance their immunogenicity and to reduce the number of doses and amount of antigen (or pathogen component) required to elicit a protective immune response, particularly in immunocompromised individuals [2,39]. To formally evaluate the adjuvant properties of Bt OMVs, mice were administered a single dose of native OMVs in PBS via the intranasal route and 5 days later head and thoracic organs were removed *en bloc* and analysed by immunohistology for the presence of organised lymphoid structures and follicles indicative of an active immune response. Large organised lymphoid follicles were present in both the nasal cavity (nasal-associated lymphoid tissue or NALT (Figure 3(a)) and the lungs (bronchus-associated lymphoid tissue or BALT) (Figure 3(b)) which contained dendritic cells, T cells and large numbers of B cells. These structures were absent in mice administered PBS alone (Figure 3(a,b)). Of note, OMVs were also effective at eliciting the formation of lymphoid clusters in mediastinal adipose tissue (fat-associated lymphoid clusters or FALC) (Figure 3(c)). Consistent with the immune response priming ability of Bt OMVs, within 24 h of intranasal administration of fluorescent labelled native OMVs it was possible to detect their uptake in the NALT and draining cervical lymph nodes (CLN) (Supplementary Fig. S1). At day 5, there was evidence of trafficking of OMVs to both the cervical and mesenteric lymph nodes which was almost exclusively mediated by CD11c^+^, CD11b^+^ CD103^−^ dendritic cells (Supplementary Figure S1).

From a biosafety perspective, neither orally nor intransally administered native OMVs or vaccine antigen formulated OMVs had any adverse health effects with no tissue pathology evident in treated animals at post mortem (data not shown). Orally administered OMVs also had no or a minor and/or transient effects on intestinal microbes as determined from culturing faecal samples on selective media (Supplementary Fig. S2).

### Mucosal delivery of OMV vaccine formulations

**Figure 4.**
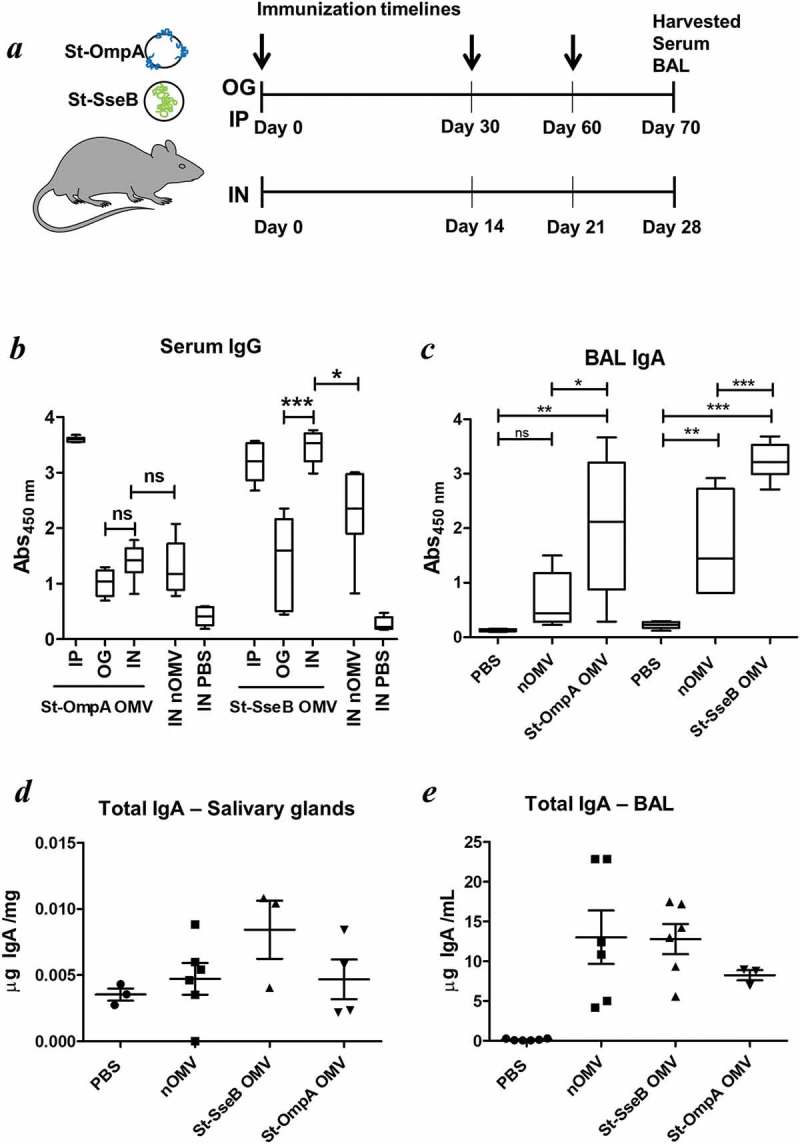
Bt OMV-elicitied systemic and mucosal antibody responses. (a) Mice (n = 5–6/grp) were administered Bt OMVs expressing the *Salmonella* OmpA or SseB proteins via the oral (OG), intranasal (IN) or intraperitoneal (IP) routes according to the dosing regimen described in the Material and Methods section. Arrows indicate time of immunization. Naïve OMVs (nOMV) and PBS were administrated to mice (n = 5–6/grp) as control groups. At autopsy, serum (b) and brochoalveolar lavage fluid (BAL) (c) were analysed for anti-OmpA and anti-SseB IgG and IgA antibody titres, respectively, by ELISA. The boxplots indentify the mean and upper and lower quartile values for data sets obtained from animals within each treatment group. Analysis of variance for multiple comparisons of means between independent samples (ANOVA) was followed by a Tukey’s test. *P < 0.05; **P < 0.01; ***P < 0.001; ns,not significant. Total IgA levels were also determined in salivary gland tissue homogenates (d) and in BAL (e) samples from each group of animals by ELISA using IgA standards as described in the Materials and Methods section.

We used preparations of OMVs expressing *Salmonella* vaccine antigen (St-OmpA and St-SseB) to compare and optimise the effectiveness of different formulations and routes of administration for the generation of mucosal and systemic antibody responses. St-OmpA and St-SseB expressing OMVs were administered to mice via the oral or nasal routes (Figure 4(a)) and also parenterally for comparison. At the end of the study BAL and serum samples were analysed by ELISA for antigen-specific IgA (mucosal) and IgG (serum) antibodies, respectively. For St-OmpA OMVs the intraperitoneal route of administration generated the highest levels of antigen-specific serum IgG antibodies compared with the oral or intranasal routes of delivery, for which there were comparable, but only low levels, of St-OmpA-specific IgG (Figure 4(b)). For St-SseB OMVs, the intranasal route of administration generated significantly higher levels of antigen-specific serum IgG antibodies compared with oral delivery and were equivalent to the levels of antigen-specific IgG seen after intraperitoneal immunisation (Figure 4(b)).

St-OmpA and St-SseB OMVs were equally effective at elicting antigen specific IgA antibodies in the lower respiratory tract and BAL (Figure 4(c)). However, there was more individual variation in the IgA levels and response to St-OmpA OMVs compared with animals administered St-SseB OMVs. Intranasally-administered St-OmpA and St-SseB OMVs also increased global IgA antibody production and secretion in both the salivary glands (Figure 4(d)) and BAL (Figure 4(e)). Of note, naïve OMVs also increased global IgA levels in these sites (Figure 4(d,e)), which is consistent with the adjuvant properties of Bt OMVs and their ability to activate the immune system in both of these sites, and generate organised lymphoid follicles and tissues containing large numbers of B cells (Figure 3).

Despite the induction of *Salmonella* antigen-specific IgG antibodies, neither oral nor parenteral vaccination with StOmpA-OMVs conferred signficiant levels of protection to oral *Salmonella* infection as judged by the pathogen burden (CFU) in intestinal and extra-intestinal tissues at 5 days post infection (Supplementary Fig. S3).

### Intranasal OMV viral vaccine formulations protect against pulmonary IAV infection

Based on the potent adjuvant effect of intranasally administered OMVs (Figure 3) and higher levels of antigen specific mucosal and systemic antigen specific antibodies after intranasal immunisation with OMV based vaccines (Figure 4), we used IAV vaccine formulated OMVs to investigate further the possibility that intranasal immunisation with OMV-based vaccines is a better option for mucosal vaccination and conferring protection to infectious challenge.

**Figure 5.**
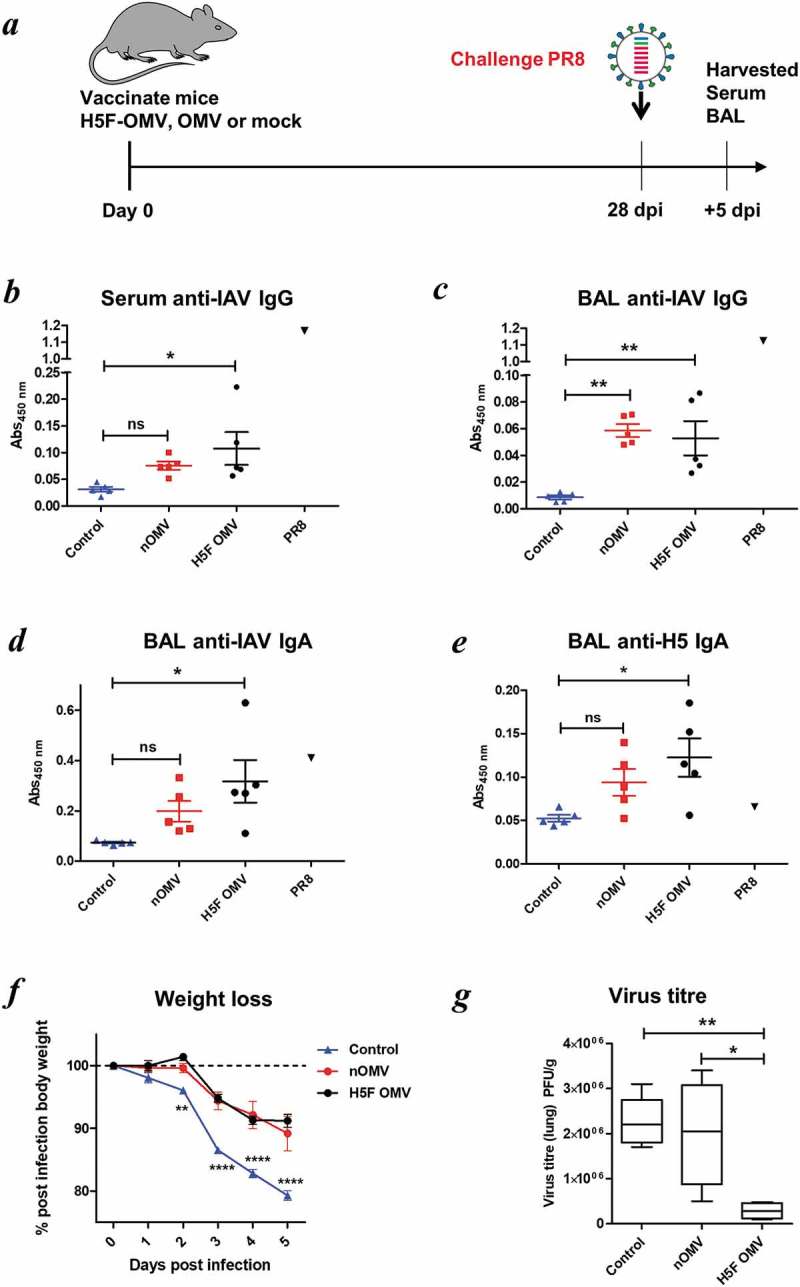
Bt OMVs expressing IAV H5F protein confer a level of protection to virus infection in mice. (a) Mice were immunised intranasally with H5F-OMVs in PBS; controls were administered intranasally with naïve OMVs or PBS alone (mock) at the indicated time-points; after 28 days all were challenged intranasally with a 10-fold lethal dose of IAV strain A/PR/8/34 (PR8, H1N1). At necropsy serum (b) and brochoalveolar lavage fluid (BAL) (c, d) were analysed for IAV IgG and IgA antibodies by ELISA using UV-inactivated PR8 virus. BAL samples were also analysed for H5 HA specific IgA antibodies (e) using recombinant H5 HA as the target antigen. Immune serum and BAL from PR8 IAV-infected mice (PR8) were used as reference samples. (f) The weight of individual animals in each group was assessed daily. (g) Lung homogenates were assessed for viral load (PFU/g lung tissue) at necropsy. Statisitical analysis was performed using one-way ANOVA with Tukey’s multiple comparison tests (panels b,c,d,e,g) or two-way ANOVA with Bonferroni post-tests. ns, not significant; *P < 0.05; **P < 0.01; ***P < 0.001.

Serum anti-IAV IgG antibody levels were higher in H5F-OMV immunised animals than the other groups (Figure 5(a)). Animals immunized with naïve OMVs had levels of BAL anti-IAV IgG antibodies significantly higher than in non-immunized animals (Figure 5(b)). However, it should be noted that levels of specific IgG in the OMV-vaccinated groups were far lower than that seen with a positive control serum from PR8-infected mice (Figure 5(b,c); black triangles). IAV specific IgA antibody levels were significantly higher in the BAL samples from animals immunised with H5F-OMVs compared with the other groups (p = 0.004) (Figure 5(d)). Of note, the levels of anti-PR8 (H1N1)-specific IgA seen in OMV-inoculated mice were comparable to those seen in the positive control BAL from homologous PR8-infected mice. Also, the levels of H5 HA-specific IgA in BAL of the control PR8-infected mice were not above control, demonstrating the lack of cross-type specific HA IgA antibodies in PR8-infected mice. Thus, vaccination with OMVs and H5F-OMVs induced levels of antibodies, in particular IgA in the BAL that were able to react with homologous H5 as well as heterotypic H1 HA molecules. During infection the weight of all infected animals declined with the greatest weight loss seen in the control (PBS administered) animals that lost almost 20% of their body weight (Figure 5(f)). Animals immunised with H5F-OMVs displayed a more gradual decline in weight loss after infection, as did those immunized with naïve OMVs. Notably, the body weight of the H5F-OMV-immunised animals stabilised with no further decline between day 4 and day 5 post-infection; this is indicative of a less severe infection from which they recovered. This was confirmed by the lung viral titre data (Figure 5(g)); viral load in animals that had been immunised with H5F-OMVs was significantly lower (p < 0.006) than that of the other groups, reflecting an approximate 7 to 8-fold lower level of virus.

### KGF-2 containing omvs protect against acute colitis

**Figure 6.**
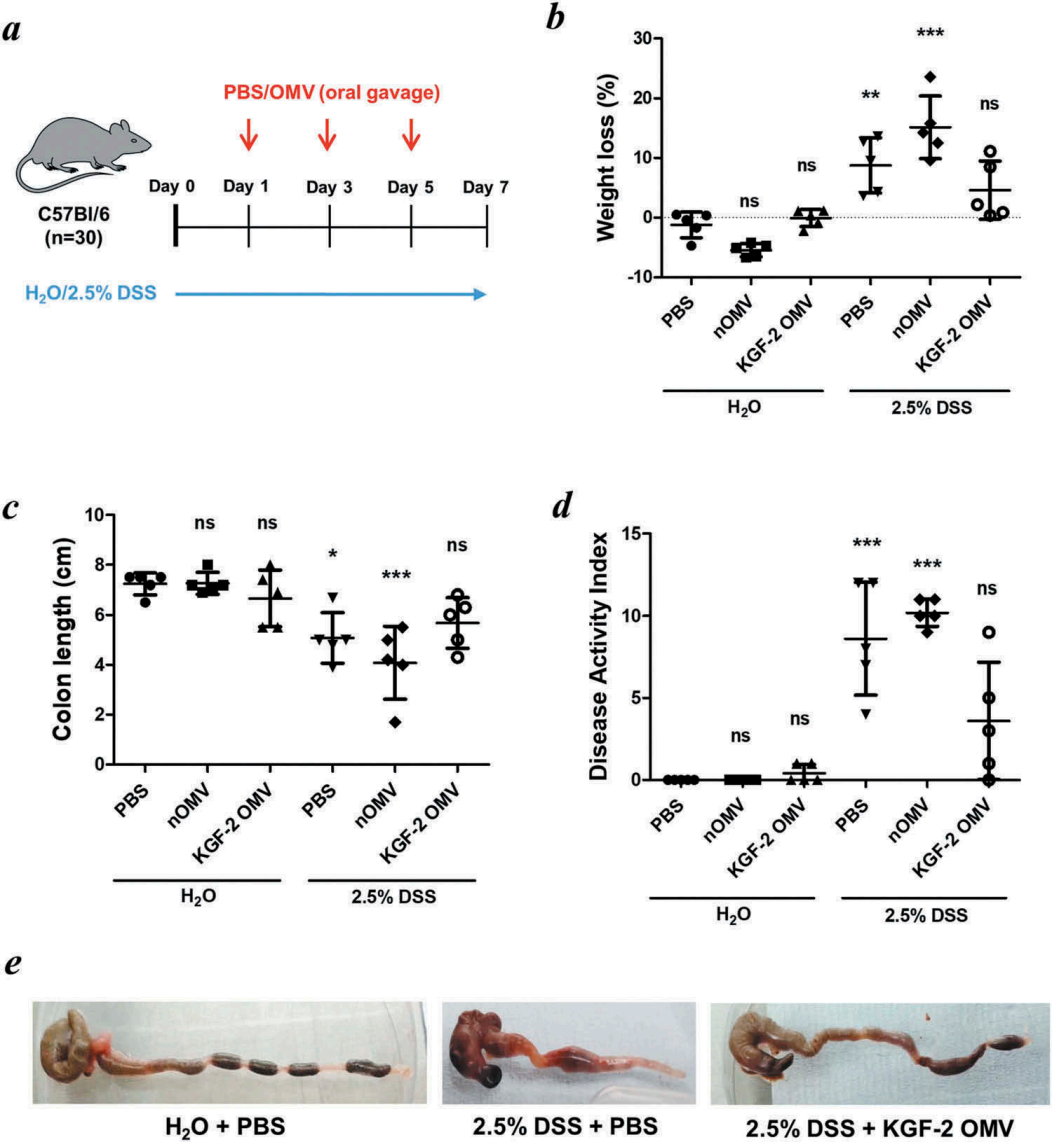
OMVs containing KGF-2 ameliorate DSS-induced colitis in mice. (a) Groups of mice were provided with drinking water with or without 2.5% (w/v) DSS for 7 days. On days 1, 3 and 5 mice were orally gavaged with either PBS, naïve OMVs or OMVs containing KGF-2. (b) Percent weight loss at day 7. (c) Colon length at day 7. (d) Disease Activity Index (DAI) at day 7. (e) Representative images of colons. Data expressed as mean ± SD (n = 5). Statisitical analysis was performed using one-way ANOVA with Tukey’s multiple comparison tests. Mice gavaged with PBS and receiving regular drinking water were considered as the reference group for statistical analysis. ns, not significant; *P < 0.05; **P < 0.01; ***P < 0.001.

To determine whether Bt OMVs were suitable for mucosal delivery of human therapeutic proteins, the KGF-2 protein was expressed in Bt OMVs. This was achieved by cloning a mini-gene containing the coding sequence of the mature human *kgf-2* gene and the Bt OmpA signal peptide in Bt (Bt-KGF-2). Immunoblotting of lysates of OMVs harvested from cultures of Bt-KGF-2 (KGF-2 OMVs) established that they contained approximately 5 µg/ml of KGF-2 (Figure 2(d)) and that the protein was contained within the lumen of the OMVs (Figure 2(c)). The biological activity of KGF-2 OMVs was confirmed in an epithelial cell wounding (scratch) assay in which the addition of intact KGF-2 OMVs to the epithelial cell cultures promoted epithelial cell proliferation and accelerated wound closure (Supplementary Fig. S4). KGF-2 OMVs were tested in the acute murine DSS colitis model (Figure 6(a)), which is a well characterised, simple and reproducible model of intestinal inflammation that is independent of lymphocyte-mediated responses and in which the clinical severity can be quantified and new therapeutic agents evaluated [40]. Since DSS primarily affects epithelial cells and inhibits their proliferation [40] this model is well suited to testing the therapeutic potential of KGF-2 OMVs. The dosing regimen was based in part on pilot experiments assessing the tolerability of OMVs (data not shown) and our previous studies using a *B. ovatus* strain engineered to express human KGF-2 *in vivo* that had a therapeutic effect in DSS-colitis [41].

**Figure 7.**
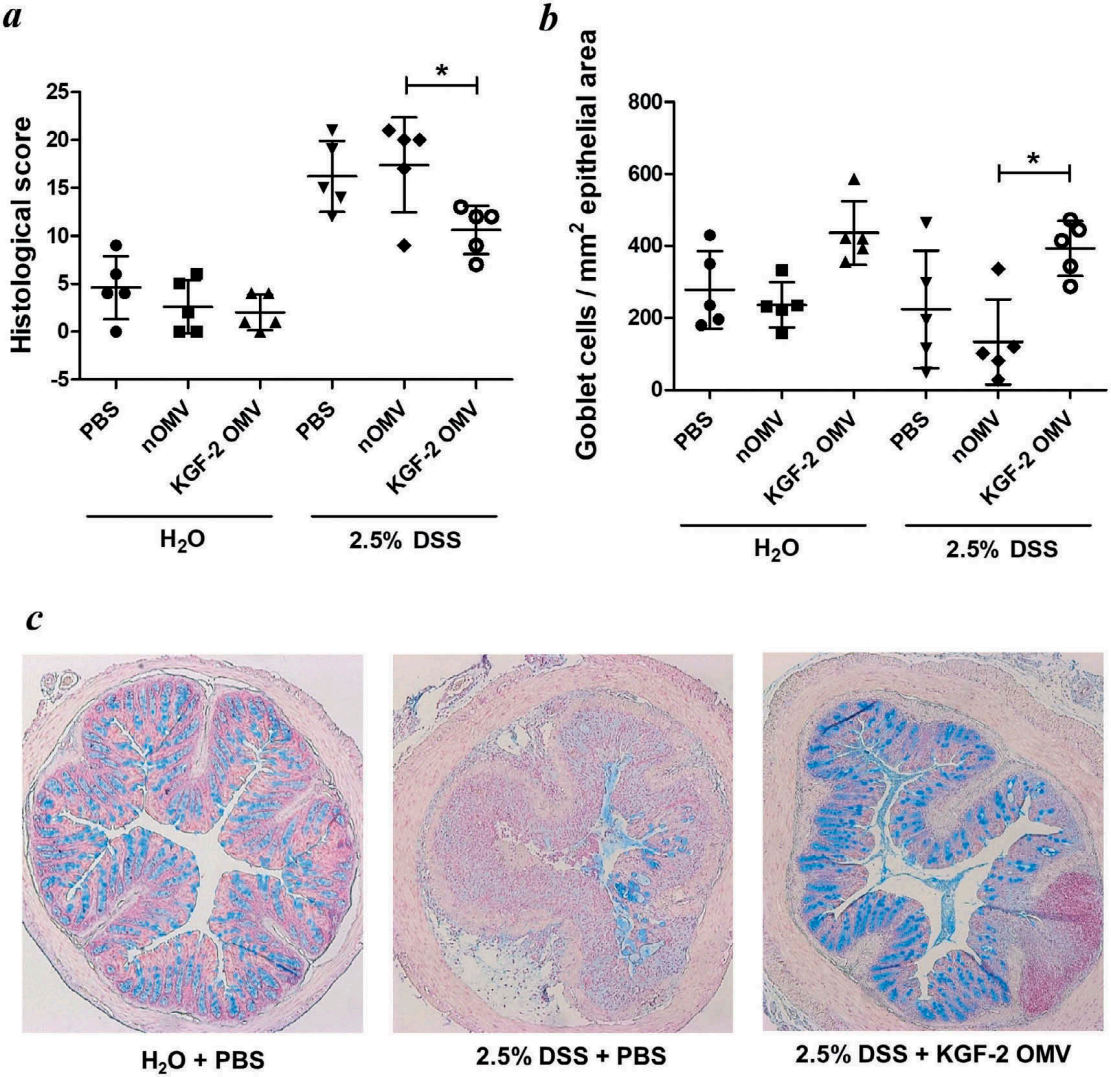
OMVs containing KGF-2 protect and restore goblet cells in mice with DSS-induced colitis. (a) Histological score of colon tissue as determined by microscopy of H&E stained sections obtained at necropsy. (b) Number of Alcian Blue stained goblet cells per mm^2^ of epithelial area. (c) Microscope images of goblet cell distribution in representative colon sections stained with Alcian Blue. Data expressed as mean ± SD (n = 5). Statisitical analysis was performed using one-way ANOVA with Tukey’s multiple comparison tests. *P < 0.05.

KGF-2 OMVs controlled colitis both clinically and pathologically. Weight loss was significantly reduced in animals receiving KGF-2 OMVs compared with non-treated animals (p < 0.01) or animals that had been administered naïve OMVs (p < 0.001) (Figure 6(b)). KGF-2 OMVs also reduced the impact of DSS on colon shrinkage and reduction in length (Figure 6(c,e)), which is an independent measure of inflammation [42]. Consistent with the therapeutic effect of KGF-2 OMVs, disease activity index scores were significantly lower in KGF-2 OMV-treated animals compared with the other treatment groups (Figure 6(d) and Supplementary Tables 1 and 2). In addition, colon shortening caused by DSS-treatment was abated by KGF-2 OMV administration (Figure 6(e)). Histopathology showed that KGF-2 OMV treatment reduced epithelial damage and inflammatory infiltrate compared with non-treated mice and mice that had been administered naïve OMVs (Figure 7(a)). KGF-2 OMVs also had a beneficial effect on mucin-producing goblet cells. Compared with non-treated animals or animals receiving naïve OMVs, there was a signficiant increase in the number of mucin-containing goblet cells in the colonic mucosa of KGF-OMV-treated animals (Figure 7(b)); the appearance and distribution of goblet cells resembled that of the control animals receiving water alone and no DSS (Figure 7(c)).

## Discussion

In this study we have provided evidence for the suitability of using OMVs from the major human gut commensal bacterium, Bt, to deliver biologics to mucosal sites and protect against infection and injury. The nanosize and non-replicative status of Bt OMVs together with their stability and ability to interact with mucosal and systemic host cells makes them ideally suited for drug delivery. Moreover, they possess innate adjuvant properties and the ability to activate immune cells and promote development of the organised mucosa-associated lymphoid follicles that are required for generating effective immune responses. The use of OMVs from prominent human commensal bacteria that have established a mutualistic relationship with, and are well tolerated by, their host is also desirable from a safety perspective and for minimising or preventing inappropriate host responses. This was evidenced by the absence of any change in health status and no pathology in Bt OMV-treated animals.

The Bt OMV technology platform is underpinned by our ability to engineer *Bacteroides* species [21,43,44] to express heterologous proteins that retain their biological activity [41,45,46]; using specific protein secretion sequences heterologous proteins can be directed to the periplasmic space for export and incorporation into the lumen or outer membrane of Bt-generated OMVs. It has not been possible to absolutely define the minimum (or optimal) level of expression of heterologous proteins necessary to elicit an appropriate host response. However, low levels of expression that are only detectable by high resolution LC-MS-based proteomics (as for *Salmonella* SseB) are sufficient to induce robust host mucosal and systemic antibody responses. For this reason there was no apparent correlation between the levels of expression of different proteins within or at the surface of OMVs, and their ability to elicit a host response. Determining the quantity of biologically active (KGF-2) protein in OMV fomulations was made difficult by their ability to resist disruption. OMV cargo does, however, become accessible after uptake by host cells and in particular, antigen presenting macrophages and dendritic cells as shown here and previously by others [8], which is most likely as a result of an OMV-intracellular membrane fusion event [12].

To date, the majority of OMV applications have focused on vaccine development [18] as they offer significant advantages over conventional vaccines; they are non-replicating, provide needle-free delivery, target mucosal sites, have an established safety record, can elicit innate and antigen-specific adaptive immune responses, possess self-adjuvant properties (i.e. microbe-associated molecular pattern molecules [MAMPs] such as LPS), and are relatively cheap and straightforward to produce. The limitations of current non-commensal and pathogen-derived OMV vaccines are the potential for unintended toxicity due to associated toxins, low expression levels of protective antigens, variable efficacy depending on source and formulation, the need for exogenous adjuvants, and the problem of incomplete protection due to strain variation. The work we describe here demonstrates that these limitations can, to a large extent, be overcome by using bioengineered Bt OMVs. The intranasal route of administration was superior to oral administration in terms of elicting high levels of *Salmonella* vaccine antigen-specific mucosal IgA and systemic IgG. This difference is most likely a reflection of anatomical differences and the ease of access to host immune inductive sites and effectiveness of aquisition by mucosa-associated antigen presenting-cells that occurs in the upper respiratory tract compared with the lower GI-tract. With smaller distances to travel in the less harsh environment of the lungs, OMVs are more readily accessible to host cells; within 24 h of intranasal administration Bt OMVs were acquired by CD103^−^ CD11b^+^ dendritic cells in the mucosa of the upper and lower respiratory tract, with some trafficking to draining lymph nodes. Importantly, this CD103^−^ CD11b^+^ population of tissue DC are known to traffic antigen to lymph nodes and initiate T cell responses [47].

The failure to demonstrate protection against infectious challenge in animals immunised with *Salmonella* OMV vaccine formulations may be as a consequence of various factors including sub-optimal expression of appropriate quantities of immunogenic St OmpA antigen in Bt OMVs and/or the generation of insufficient levels of functional, pathogen-neutralising, antibodies. Although St OmpA was previously identified as a potential cross-species vaccine candidate [48] our findings are in line with those of Okamura and colleagues [49] who found no protection in chickens parenterally immunised with St OmpA. However, the universal adjuvant properties of Bt OMVs suggests they may still be of value in *Salmonella* vaccine formulations as an adjuvant analogous to meningococcal OMVs that provide potent adjuvanticity to *N. meningitidis* recombinant protein-based vaccines [50,51].

We obtained more compelling evidence for the potential of Bt OMV-based vaccines in the IAV H5F-OMV system which, after intranasal administration, conferred a significant level of heterotypic protection against an unrelated subtype of IAV. There are numerous strains of IAV and the virus evolves rapidly. This presents a challenge to generating a “universal vaccine” that will protect against multiple IAV strains. Most IAV vaccines do not afford protection against heterologous strains of virus. Recent work has shown that while the globular head of the IAV HA molecule is highly variable and does not generate cross-protective immunity, the stem/stalk is more conserved and will generate cross-protective antibodies [52]. Naïve OMVs had a positive impact on the generation of mucosal virus-specific IgA antibodies and also reduced lung viral titres. This is most likely a reflection of their potent adjuvant properties and the activation of innate and adaptive immune responses, including raised total IgA antibody levels in the upper and lower respiratory tract which would strengthen front line protection against IAV infection [53]. At 5 days post challenge with the heterologous PR8 (H1N1) virus, the mean weight of H5F-OMV-immunized animals stabilized which is indicative of recovery from virus infection; which was supported by the fact that H5F-OMV immunized animals had a 7–8-fold lower lung viral titre compared with non-vaccinated animals or animals immunized with naïve OMVs. Future refinements to the study protocol should provide a clearer picture of the efficacy of OMV-H5F vaccines in preventing IAV infection by both homotypic and heterotypic strains of influenza virus. As our study was not an end point study we cannot directly compare the level of protection against infection conferred by OMV-H5F vaccines with similiar studies trialing OMV-based vaccines; these include those of Watkins and colleagues [54] who developed *E. coli* OMV-IAV vaccines and achieved 100% protection in a murine lethal infectious challenge model. Collectively, our data provides the rationale and justification for continuing the development and refinement of OMV technology to improve and optimse their vaccine capabilities and performance.

Our findings using KGF-2-containing OMVs to ameliorate experimental colitis demonstrates the potential for a broader portfolio of applications and, in particular, for the mucosal delivery of therapeutic proteins for the treatment of non-infectious, autoimmune-driven pathologies. The benefit of this form of drug delivery is exemplified by comparing the doses required to improve colonic pathology using OMV technology and a standard approach. The dose of KGF-2 OMVs (approximately 0.5 µg) used to achieve a significant reduction in colonic histopathology is 1–2 orders of magnitude lower than that required in daily injections (20–100 µg for 7 days) to achieve a comparable reduction in colonic pathology [55,56]. Also, the ability to deliver the protein directly to the target tissue using orally administered OMVs reduces the risk of side effects associated with systemic delivery.

In summary, our data adds to the growing number of new approaches being developed to express heterologous proteins in bacterial microvesicles [18] for a variety of applications. Our work provides evidence for the utility and effectiveness of human commensal bacteria as a source of bioengineered OMVs for the mucosal delivery of different biologics.

## References

[1] Kulp A, Kuehn MJ. Biological functions and biogenesis of secreted bacterial outer membrane vesicles. Annu Rev Microbiol. 2010;64:163–19. PubMed PMID: WOS:000284030600009; English. 20825345 10.1146/annurev.micro.091208.073413 PMC3525469

[2] Del Giudice G, Rappuoli R, Didierlaurent AM Correlates of adjuvanticity: a review on adjuvants in licensed vaccines. Semin Immunol. 2018 5 22;39:14–21. 10.1016/j.smim.2018.05.001 29801750

[3] Ellis TN, Kuehn MJ Virulence and immunomodulatory roles of bacterial outer membrane vesicles. Microbiol Mol Biol Rev. 2010 3;74(1):81–94. PubMed PMID: WOS:000275120100004; English. 20197500 10.1128/MMBR.00031-09 PMC2832350

[4] Haurat M, Elhenawy W, Feldman M Prokaryotic membrane vesicles: new insights on biogenesis and biological roles. Biol Chem. 2014;396(2):95–109. 10.1515/hsz-2014-0183 25178905

[5] Olsen I, Amano A Outer membrane vesicles - offensive weapons or good Samaritans? J Oral Microbiol. 2015;7:27468 PubMed PMID: 25840612; PubMed Central PMCID: PMCPMC4385126. 25840612 10.3402/jom.v7.27468 PMC4385126

[6] Elhenawy W, Debelyy MO, Feldman MF Preferential packing of acidic glycosidases and proteases into bacteroides outer membrane vesicles. MBio. 2014;5(2):e00909–e00914. PubMed PMID: 24618254; PubMed Central PMCID: PMC3952158. 24618254 10.1128/mBio.00909-14 PMC3952158

[7] Rakoff-Nahoum S, Coyne MJ, Comstock LE An ecological network of polysaccharide utilization among human intestinal symbionts. Curr Biol. 2014 1 6;24(1):40–49. PubMed PMID: 24332541; PubMed Central PMCID: PMC3924574. 24332541 10.1016/j.cub.2013.10.077 PMC3924574

[8] Stentz R, Osborne S, Horn N, et al A bacterial homolog of a eukaryotic inositol phosphate signaling enzyme mediates cross-kingdom dialog in the mammalian gut. Cell Rep. 2014 2 27;6(4):646–656. PubMed PMID: 24529702; PubMed Central PMCID: PMC3969271. 24529702 10.1016/j.celrep.2014.01.021 PMC3969271

[9] Bryant WA, Stentz R, Le Gall G, et al In silico analysis of the small molecule content of outer membrane vesicles produced by Bacteroides thetaiotaomicron indicates an extensive metabolic link between microbe and host. Front Microbiol. 2017;8:2440 PubMed PMID: 29276507; PubMed Central PMCID: PMCPMC5727896. 29276507 10.3389/fmicb.2017.02440 PMC5727896

[10] Shen Y, Giardino Torchia ML, Lawson GW, et al Outer membrane vesicles of a human commensal mediate immune regulation and disease protection. Cell Host Microbe. 2012 10 18;12(4):509–520. PubMed PMID: 22999859; PubMed Central PMCID: PMC3895402. 22999859 10.1016/j.chom.2012.08.004 PMC3895402

[11] Hickey CA, Kuhn KA, Donermeyer DL, et al Colitogenic Bacteroides thetaiotaomicron antigens access host immune cells in a sulfatase-dependent manner via outer membrane vesicles. Cell Host Microbe. 2015 5 13;17(5):672–680. PubMed PMID: 25974305; PubMed Central PMCID: PMC4432250. 25974305 10.1016/j.chom.2015.04.002 PMC4432250

[12] Kaparakis-Liaskos M, Ferrero RL Immune modulation by bacterial outer membrane vesicles. Nat Rev Immunol. 2015 6;15(6):375–387. PubMed PMID: 25976515. 25976515 10.1038/nri3837

[13] Stentz R, Carvalho AL, Jones EJ, et al Fantastic Voyage: the journey of intestinal microbiota-derived microvesicles through the body. Biochem Soc Trans. 2018;46(5):1012-1-27. 10.1042/BST20180114 PMC6195637 30154095

[14] Kesty NC, Kuehn MJ Incorporation of heterologous outer membrane and periplasmic proteins into Escherichia coli outer membrane vesicles. J Biol Chem. 2004 1 16;279(3):2069–2076. PubMed PMID: WOS:000188005700062; English. 14578354 10.1074/jbc.M307628200 PMC3525464

[15] Collins BS Gram-negative outer membrane vesicles in vaccine development. Discov Med. 2011 7;62:7–15. PubMed PMID: WOS:000208639700001; English. 21794204

[16] de Kleijn ED, de Groot R, Labadie J, et al Immunogenicity and safety of a hexavalent meningococcal outer-membrane-vesicle vaccine in children of 2-3 and 7-8 years of age. Vaccine. 2000 2 14;18(15):1456–1466. PubMed PMID: WOS:000085195800005; English. 10618543 10.1016/s0264-410x(99)00423-5

[17] Sandbu S, Feiring B, Oster P, et al Immunogenicity and safety of a combination of two serogroup B meningococcal outer membrane vesicle vaccines. Clin Vaccin Immunol. 2007 9;14(9):1062–1069. PubMed PMID: WOS:000249488400002; English. 10.1128/CVI.00094-07 PMC2043307 17634513

[18] Gerritzen MJH, Martens DE, Wijffels RH, et al Bioengineering bacterial outer membrane vesicles as vaccine platform. Biotechnol Adv. 2017 9;35(5):565–574. PubMed PMID: 28522212. 28522212 10.1016/j.biotechadv.2017.05.003

[19] Shoemaker NB, Getty C, Gardner JF, et al Tn4351 transposes in bacteroides spp and mediates the integration of plasmid R751 into the Bacteroides chromosome. J Bacteriol. 1986 3;165(3):929–936. PubMed PMID: WOS:A1986A276700039; English. 3005243 10.1128/jb.165.3.929-936.1986 PMC214518

[20] Stentz R, Horn N, Cross K, et al Cephalosporinases associated with outer membrane vesicles released by bacteroides spp. protect gut pathogens and commensals against beta-lactam antibiotics. J Antimicrob Chemother. 2015 3;70(3):701–709. PubMed PMID: 25433011; PubMed Central PMCID: PMC4319488. 25433011 10.1093/jac/dku466 PMC4319488

[21] Wegmann U, Horn N, Carding SR Defining the bacteroides ribosomal binding site. Appl Environ Microbiol. 2013 3;79(6):1980–1989. PubMed PMID: WOS:000315454500026; English. 23335775 10.1128/AEM.03086-12 PMC3592243

[22] Wegmann U, Klein JR, Drumm I, et al Introduction of peptidase genes from Lactobacillus delbrueckii subsp. lactis into Lactococcus lactis and controlled expression. Appl Environ Microbiol. 1999 11;65(11):4729–4733. PubMed PMID: 10543778; PubMed Central PMCID: PMCPMC91636. 10543778 10.1128/aem.65.11.4729-4733.1999 PMC91636

[23] Matrosovich M, Matrosovich T, Garten W, et al New low-viscosity overlay medium for viral plaque assays. Virol J. 2006 8 31;3:63 PubMed PMID: 16945126; PubMed Central PMCID: PMCPMC1564390. 16945126 10.1186/1743-422X-3-63 PMC1564390

[24] Stewart JP, Kipar A, Cox H, et al Induction of tachykinin production in airway epithelia in response to viral infection. PLoS One. 2008;3(3):e1673 PubMed PMID: 18320026; PubMed Central PMCID: PMC2248620. eng. 18320026 10.1371/journal.pone.0001673 PMC2248620

[25] Schmid AS, Hemmerle T, Pretto F, et al Antibody-based targeted delivery of interleukin-4 synergizes with dexamethasone for the reduction of inflammation in arthritis. Rheumatology (Oxford). 2018 4 1;57(4):748–755. PubMed PMID: 29365185; PubMed Central PMCID: PMCPMC6104808. 29365185 10.1093/rheumatology/kex447 PMC6104808

[26] Gil-Cruz C, Bobat S, Marshall JL, et al The porin OmpD from nontyphoidal Salmonella is a key target for a protective B1b cell antibody response. Proc Natl Acad Sci U S A. 2009 6 16;106(24):9803–9808. PubMed PMID: WOS:000267045500047; English. 19487686 10.1073/pnas.0812431106 PMC2701014

[27] Kurtz JR, Petersen HE, Frederick DR, et al Vaccination with a single CD4 T cell peptide epitope from a Salmonella type III-secreted effector protein provides protection against lethal infection. Infect Immun. 2014 6;82(6):2424–2433. PubMed PMID: WOS:000336378100029; English. 24686055 10.1128/IAI.00052-14 PMC4019158

[28] Barat S, Willer Y, Rizos K, et al Immunity to intracellular Salmonella depends on surface-associated antigens. PLoS Pathog. 2012 10;8(10):e1002966 PubMed PMID: WOS:000310530300029; English. 23093937 10.1371/journal.ppat.1002966 PMC3475680

[29] Rollenhagen C, Sorensen M, Rizos K, et al Antigen selection based on expression levels during infection facilitates vaccine development for an intracellular pathogen. Proc Natl Acad Sci U S A. 2004 6 8;101(23):8739–8744. PubMed PMID: WOS:000222037000045; English. 15173591 10.1073/pnas.0401283101 PMC423265

[30] McSorley SJ, Cookson BT, Jenkins MK Characterization of CD4(+) T cell responses during natural infection with Salmonella typhimurium. J Iimmunol. 2000 1 15;164(2):986–993. PubMed PMID: WOS:000084708600057; English. 10.4049/jimmunol.164.2.986 10623848

[31] Paramasivam N, Linke D ClubSub-P: cluster-based subcellular localization prediction for Gram-negative bacteria and archaea. Front Microbiol. 2011;2:218 PubMed PMID: WOS:000208863500226; English. 22073040 10.3389/fmicb.2011.00218 PMC3210502

[32] Burton NA, Schurmann N, Casse O, et al Disparate impact of oxidative host defenses determines the fate of Salmonella during systemic infection in mice. Cell Host Microbe. 2014 1 15;15(1):72–83. PubMed PMID: WOS:000330854100010; English. 24439899 10.1016/j.chom.2013.12.006

[33] Lee SJ, McLachlan JB, Kurtz JR, et al Temporal expression of bacterial proteins instructs host cd4 t cell expansion and Th17 development. PLoS Pathog. 2012 1;8(1):e1002499 PubMed PMID: WOS:000300767100039; English. 22275869 10.1371/journal.ppat.1002499 PMC3262010

[34] Reynolds CJ, Jones C, Blohmke CJ, et al The serodominant secreted effector protein of Salmonella, SseB, is a strong CD4 antigen containing an immunodominant epitope presented by diverse HLA class II alleles. Immunology. 2014 11;143(3):438–446. PubMed PMID: WOS:000342894300013; English. 24891088 10.1111/imm.12327 PMC4212957

[35] Mallajosyula VV, Citron M, Ferrara F, et al Influenza hemagglutinin stem-fragment immunogen elicits broadly neutralizing antibodies and confers heterologous protection. Proc Natl Acad Sci U S A. 2014 6 24;111(25):E2514–E2523. PubMed PMID: 24927560; PubMed Central PMCID: PMCPMC4078824. 24927560 10.1073/pnas.1402766111 PMC4078824

[36] Valkenburg SA, Mallajosyula VV, Li OT, et al Stalking influenza by vaccination with pre-fusion headless HA mini-stem. Sci Rep. 2016 3 7;6:22666 PubMed PMID: 26947245; PubMed Central PMCID: PMCPMC4780079. 26947245 10.1038/srep22666 PMC4780079

[37] Werner S Keratinocyte growth factor: a unique player in epithelial repair processes. Cytokine Growth Factor Rev. 1998 6;9(2):153–165. PubMed PMID: 9754709. 9754709 10.1016/s1359-6101(98)00010-0

[38] Baumgart DC, Sandborn WJ Inflammatory bowel disease: clinical aspects and established and evolving therapies. Lancet. 2007 5 12;369(9573):1641–1657. PubMed PMID: 17499606. 17499606 10.1016/S0140-6736(07)60751-X

[39] Saroja C, Lakshmi P, Bhaskaran S Recent trends in vaccine delivery systems: a review. Int J Pharm Investig. 2011 4;1(2):64–74. PubMed PMID: 23071924; PubMed Central PMCID: PMCPMC3465129. 10.4103/2230-973X.82384 PMC3465129 23071924

[40] Dieleman LA, Ridwan BU, Tennyson GS, et al Dextran sulfate sodium-induced colitis occurs in severe combined immunodeficient mice. Gastroenterology. 1994 12;107(6):1643–1652. PubMed PMID: 7958674. 7958674 10.1016/0016-5085(94)90803-6

[41] Hamady ZZ, Scott N, Farrar MD, et al Xylan-regulated delivery of human keratinocyte growth factor-2 to the inflamed colon by the human anaerobic commensal bacterium Bacteroides ovatus. Gut. 2010 4;59(4):461–469. PubMed PMID: 19736360. 19736360 10.1136/gut.2008.176131

[42] Okayasu I, Hatakeyama S, Yamada M, et al A novel method in the induction of reliable experimental acute and chronic ulcerative colitis in mice. Gastroenterology. 1990;98:694–702. 1688816 10.1016/0016-5085(90)90290-h

[43] Hamady ZZ, Farrar MD, Whitehead TR, et al Identification and use of the putative Bacteroides ovatus xylanase promoter for the inducible production of recombinant human proteins. Microbiology. 2008 10;154(Pt 10):3165–3174. PubMed PMID: 18832322. 18832322 10.1099/mic.0.2008/019109-0

[44] Wegmann U, Carvalho AL, Stocks M, et al Use of genetically modified bacteria for drug delivery in humans: revisiting the safety aspect. Sci Rep. 2017 5 23;7(1):2294 PubMed PMID: 28536456; PubMed Central PMCID: PMCPMC5442108. 28536456 10.1038/s41598-017-02591-6 PMC5442108

[45] Farrar M, Whithead TR, Lan J, et al Engineering of the gut commensal bacterium *Bacteroides ovatus* to produce and secrete biologically active murine interluekin-2 in response to xylan. J Appl Microbiol. 2005;98:1191–1197. 15836489 10.1111/j.1365-2672.2005.02565.x

[46] Hamady ZZ, Scott N, Farrar MD, et al Treatment of colitis with a commensal gut bacterium engineered to secrete human TGF-beta1 under the control of dietary xylan 1. Inflamm Bowel Dis. 2011 9;17(9):1925–1935. PubMed PMID: 21830271. 21830271 10.1002/ibd.21565

[47] Ballesteros-Tato A, Leon B, Lund FE, et al Temporal changes in dendritic cell subsets, cross-priming and costimulation via CD70 control CD8(+) T cell responses to influenza. Nat Immunol. 2010 3;11(3):216–224. PubMed PMID: 20098442; PubMed Central PMCID: PMCPMC2822886. 20098442 10.1038/ni.1838 PMC2822886

[48] Brandtzaeg P Gate-keeper function of the intestinal epithelium. Benef Microbes. 2013 3 1;4(1):67–82. PubMed PMID: 23257015. 23257015 10.3920/BM2012.0024

[49] Okamura M, Ueda M, Noda Y, et al Immunization with outer membrane protein A from Salmonella enterica serovar Enteritidis induces humoral immune response but no protection against homologous challenge in chickens. Poult Sci. 2012 10;91(10):2444–2449. PubMed PMID: 22991526. 22991526 10.3382/ps.2012-02303

[50] Sanders H, Feavers IM Adjuvant properties of meningococcal outer membrane vesicles and the use of adjuvants in Neisseria meningitidis protein vaccines. Expert Rev Vaccines. 2011 3;10(3):323–334. PubMed PMID: 21434800. 21434800 10.1586/erv.11.10

[51] Moshiri A, Dashtbani-Roozbehani A, Najar Peerayeh S, et al Outer membrane vesicle: a macromolecule with multifunctional activity. Hum Vaccin Immunother. 2012 7;8(7):953–955. PubMed PMID: 22699443. 22699443 10.4161/hv.20166

[52] Krammer F, Palese P Advances in the development of influenza virus vaccines. Nat Rev Drug Discov. 2015 3;14(3):167–182. PubMed PMID: 25722244. 25722244 10.1038/nrd4529

[53] Renegar KB, Small PA Jr., Boykins LG, et al Role of IgA versus IgG in the control of influenza viral infection in the murine respiratory tract. J Immunol. 2004 8 1;173(3):1978–1986. PubMed PMID: 15265932. 15265932 10.4049/jimmunol.173.3.1978

[54] Watkins HC, Rappazzo CG, Higgins JS, et al Safe recombinant outer membrane vesicles that display M2e elicit heterologous influenza protection. Mol Ther. 2017 4 5;25(4):989–1002. PubMed PMID: 28215994; PubMed Central PMCID: PMCPMC5383554. 28215994 10.1016/j.ymthe.2017.01.010 PMC5383554

[55] Zeeh JM, Procaccino F, Hoffmann P, et al Keratinocyte growth factor ameliorates mucosal injury in an experimental model of colitis in rats. Gastroenterology. 1996 4;110(4):1077–1083. PubMed PMID: 8612996. 8612996 10.1053/gast.1996.v110.pm8612996

[56] Miceli R, Hubert M, Santiago G, et al Efficacy of keratinocyte growth factor-2 in dextran sulfate sodium-induced murine colitis. J Pharmacol Exp Ther. 1999 7;290(1):464–471. PubMed PMID: 10381813. 10381813

