# Supplementary material for: Bioengineering commensal bacteria-derived outer membrane vesicles for delivery of biologics to the gastrointestinal and respiratory tract
Source: J Extracell Vesicles. 2019 Jun 24;8(1):1632100. doi: 10.1080/20013078.2019.1632100 (PMC6598475; doi:10.1080/20013078.2019.1632100)
Supplement: Supplemental Material [file ZJEV_A_1632100_SM7381.zip › 1632100_Supplementary Files/supplementary legend and table.docx]

**Supplementary Data**

**Figure S1**: Acquisition of fluorescent labelled Bt OMVs by dendritic cells and and subsequent trafficking to lymph nodes after intranasal adminstration. OMVs were fluorescently-labelled with DiO and administered intranasally to mice. At d1 and d5 post-inoculation mice were sacrificed. Cells from nasal-associated lymphoid tissue (NALT), cervical lymph nodes (CLN) and mediastinal lymph nodes (MLN) were separated and analysed by FACS using antibodies to dendritic cells (CD11c) and antibodies to define dendritic cell subsets (CD11b and CD103). (a) Representative gating strategy showing from left to right gating out debris, for single cells, for total dendritic cells (DC, CD11c^+^), for the subset of DC that have taken up OMV (DiO^+^, CD11c^+^) and the for the subsets of OMV^+^ DC that express either CD11b or CD103. (b) Day 1 post-administration. Graphs show the percentage of cells in NALT and CLN that are DCs, and the percentage of DC that are labelled or labelled and CD11b^+^. (c) Day 5 post-administration. Graphs show the percentage of cells in CLN and MLN that are DCs, and the percentage of DC that are labelled or labelled and CD11b^+^ or CD103^+^.

**Figure S2**: The impact of orally administered Bt OMVs on the recipients’ intestinal microbiota. Mice (n=5) were orally gavaged with naïve OMVs on day 3, 5 and 7, and feces was collected on day 0, 4, 7 and 8 to evaluate the impact of OMVs on the host microbiota. Feces were weighed, homogenized in PBS and serially diluted prior to plating on different agar media to select for: Total aerobes (on nutrient media); Total anaerobes (on Wilkins-Chalgren [W-G] or Brain Heart Infusion [BHI] media); or *Bacteroides* spp. (on BHI media supplemented with gentamicin and amikacin). The results are expressed in the logarithm of the CFU normalized to the weight of individual faecal samples for each day and growth medium. Data are expressed as means ± SD. Statistically significant differences were evaluated using a Dunnett bilateral post-hoc test to compare days after OMV administration vs. the control day 0. *P<0.05; **P<0.01.

**Figure S3**: Colonization of OMV-StOmpA immunised mice after *Salmonella* challenge. Mice (n = 5-6/grp) immunised with either naïve Bt OMVs or Bt StOmpA-OMVs via the oral or parental (intraperitoneal; IP.) route (see Materials and Methods for immunisation protocol) were subsequently challenged with an oral dose of 10^8^ CFU *S.* *enterica* ser. Typhimurium SL1344. Five days later animals were euthanised and the bacterial load in the ileum and colonic contents; homogenates of mesenteric lymph nodes (MLN); ileum tissue; spleen; and liver were determined by plating serial dilutions onto xylose lysine deoxycholate agar plates supplemented with 50 µg/ml streptomycin. The boxplots indentify the mean and upper and lower quartile values for data sets obtained from animals within each treatment group.

**Figure S4**: Evaluating the biological activity of KGF-2 contained in Bt OMVs using an epithelial wound-healing assay. (a) Representative micrographs of healing of a scratch wound in a confluent monolayer of Caco2 cells after exposure to PBS, naïve OMVs, KGF-2 OMVs or recombinant KGF-2 for 72h. Red dotted lines demarcate the wound margin. (b) Graphical representation of cell growth across the wound area after 72h as determined by pixel^2^.

**Table S1**: Disease Activity Index (DAI) criteria and scoring

| **Weight loss** | **Stool consistency** | **Bleeding** | **Appearance of caecum & colon** | **Appearance of caecum & colon contents** | **Score** |
| --- | --- | --- | --- | --- | --- |
| <1% | Well-formed pellets | None | Normal | Regular shape | 0 |
| 1-5% |  |  | White, abnormal size, strictures | Irregular but formed | 1 |
| 6-10% | Loose | Slight |  | Random shape | 2 |
| 11-15% |  |  |  | Blood in colon | 3 |
| >15% | Diarrhoea | Gross |  | Blood in caecum | 4 |

**Table S2**: Colon histology scoring

| **Category** |  | **Criteria** | **Score** |
| --- | --- | --- | --- |
| Inflammatory cell infiltrate | Severity | No infiltration | 0 |
|  |  | Minimal 0-10 % | 1 |
|  |  | Mild 11-25 % | 2 |
|  |  | Moderate 26-50 % | 3 |
|  |  | Marked > 51 % | 4 |
|  | Extent | No infiltration | 0 |
|  |  | Mucosal | 1 |
|  |  | Mucosal and submucosal | 2 |
|  |  | Mucosal, submucosal and transmural | 3 |
| Presence of oedema | Extent | No oedema | 0 |
|  |  | in 0 to 25 % of the section | 1 |
|  |  | in 26 to 50 % of the section | 2 |
|  |  | in more than 51 % of the section | 3 |
| Epithelial changes | Goblet cell loss | None or increase | 0 |
|  |  | Minimal: 0-20 % | 2 |
|  |  | Mild: 21-35 % | 3 |
|  |  | Moderate: 36-50 % | 4 |
|  |  | Marked: > 50 % | 5 |
|  | Erosion | Absence | 0 |
|  |  | Presence | 1 |
| Mucosal architecture | Extent | Irregular crypts | 4 |
|  |  | Crypt loss | 5 |

**Table S3**: Proteomic analysis of OMV-SseB treated or non-treated with Proteinase K

| **OMV-SseB minus PK** | **OMV-SseB plus PK** |
| --- | --- |
| DNGILIDGMTIDDYMAK | DNGILIDGMTIDDYMAK |
|  | EEVPEDVIK |
| FIEVQK | FIEVQK |
| LDKGGLQAIK | LDKGGLQAIK |
|  | MSQELNAVLTQLTGLISK |
|  | MSSGNILWGSQNPIVFK |
| NTDLMSQGQITIQK | NTDLMSQGQITIQK |
| SNEMDEVIAK | SNEMDEVIAK |
| TKEEVPEDVIK | TKEEVPEDVIK |
|  | WGEISSMIAQK |
