# Supplementary figures and images for: Bioengineering commensal bacteria-derived outer membrane vesicles for delivery of biologics to the gastrointestinal and respiratory tract
Source: J Extracell Vesicles. 2019 Jun 24;8(1):1632100. doi: 10.1080/20013078.2019.1632100 (PMC6598475; doi:10.1080/20013078.2019.1632100)

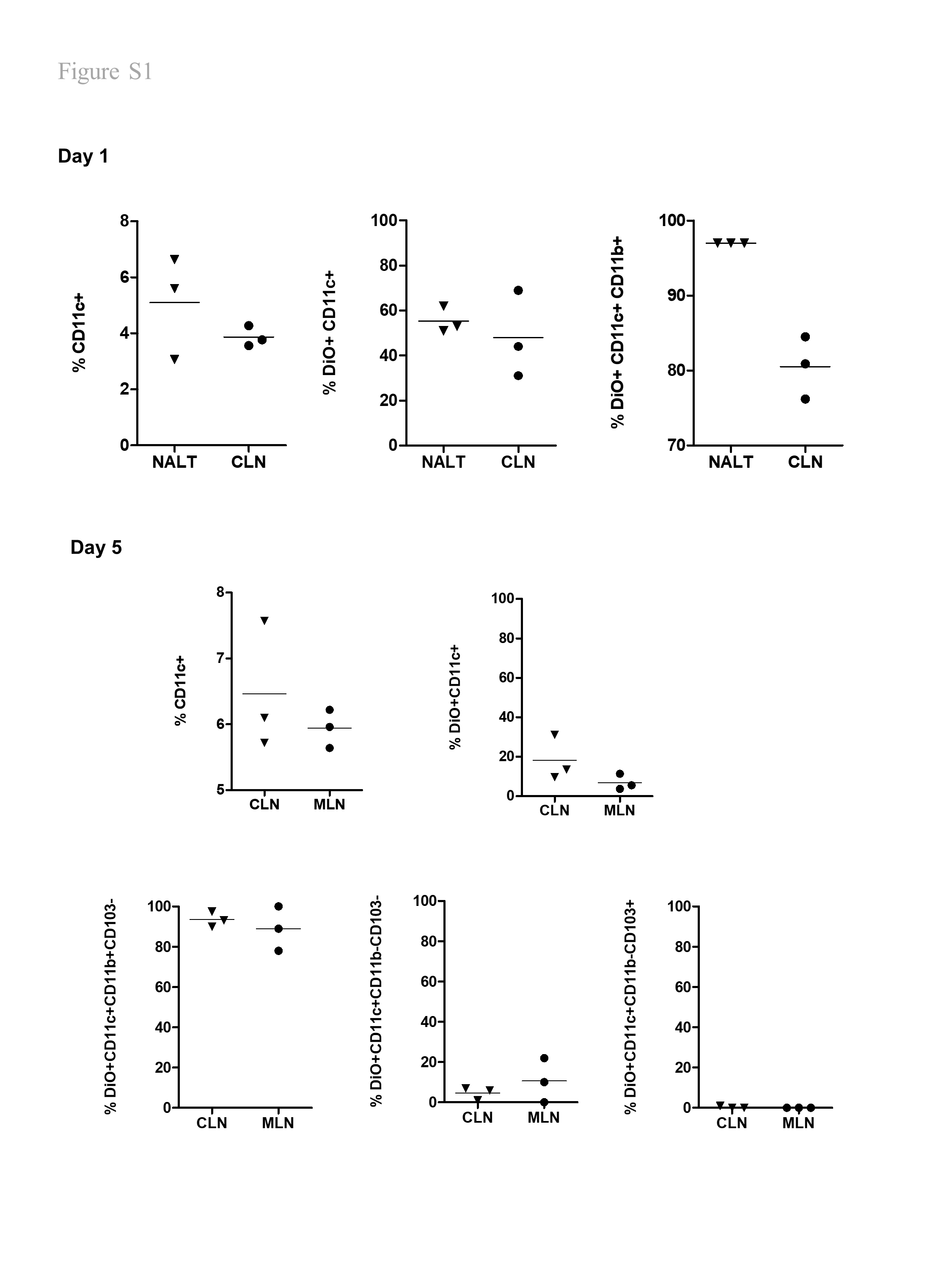

Supplement: Supplemental Material [file ZJEV_A_1632100_SM7381.zip › 1632100_Supplementary Files/Figure S1.tif]

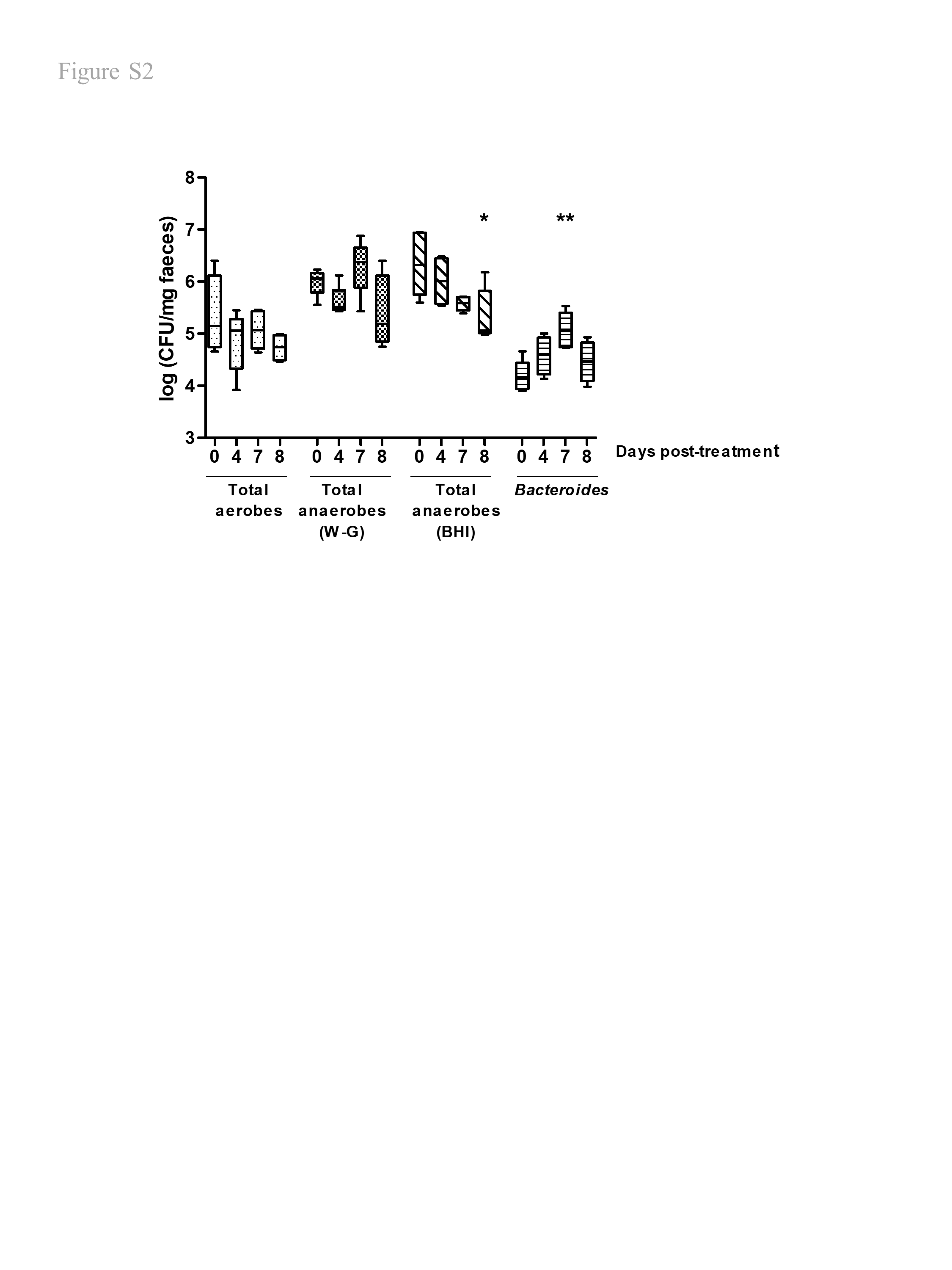

Supplement: Supplemental Material [file ZJEV_A_1632100_SM7381.zip › 1632100_Supplementary Files/Figure S2.tif]

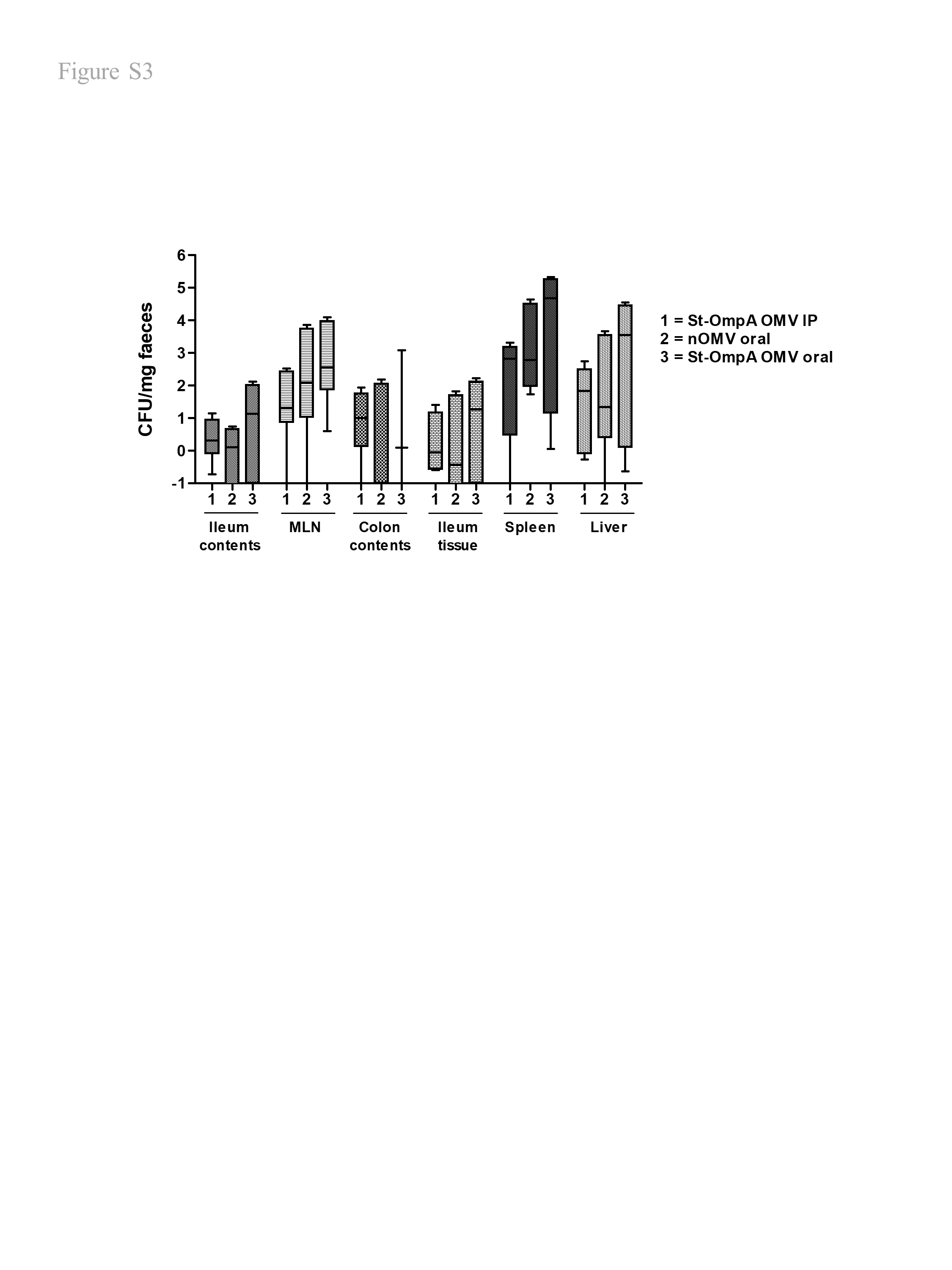

Supplement: Supplemental Material [file ZJEV_A_1632100_SM7381.zip › 1632100_Supplementary Files/Figure S3.tif]

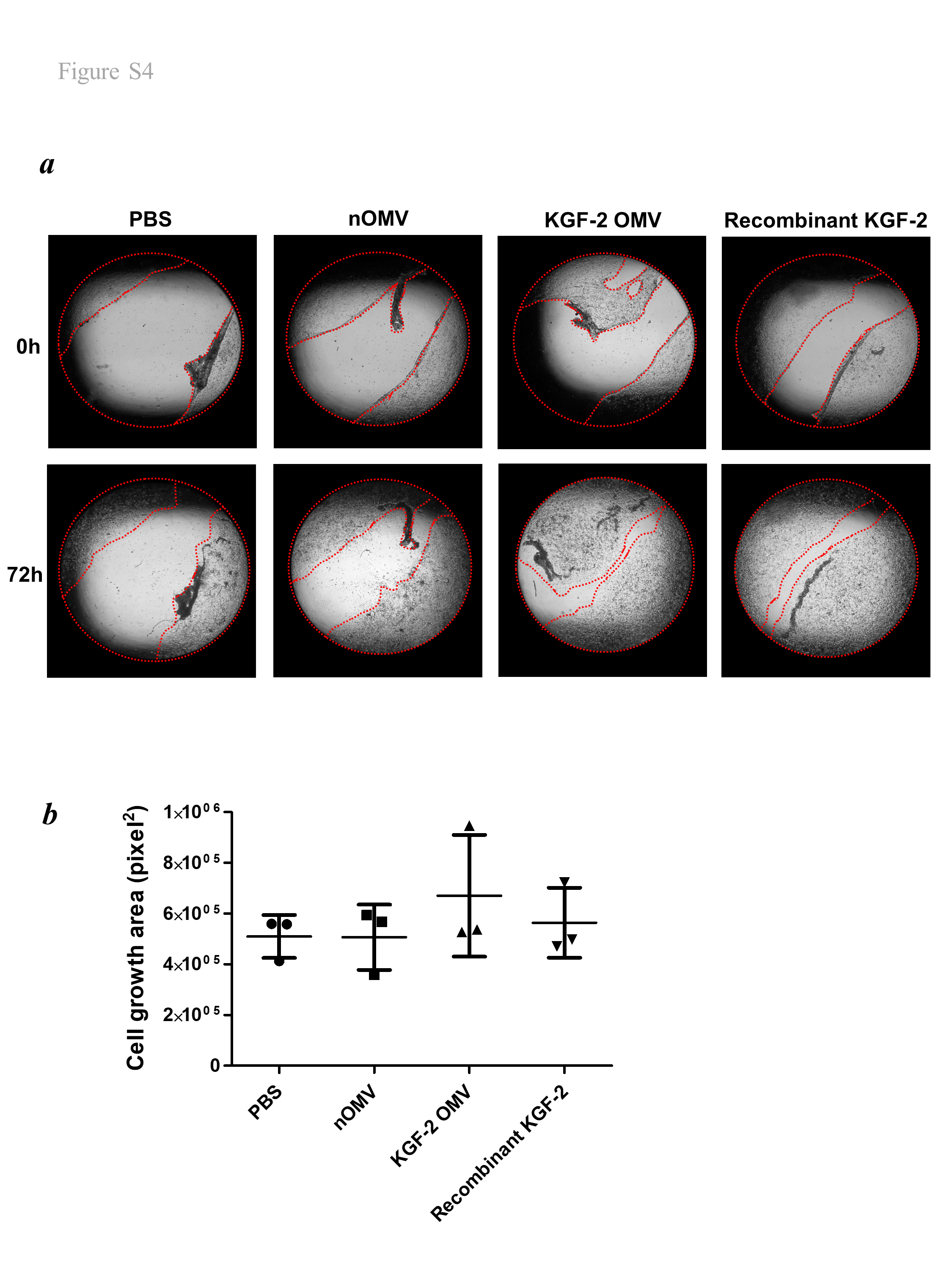

Supplement: Supplemental Material [file ZJEV_A_1632100_SM7381.zip › 1632100_Supplementary Files/Figure S4.tif]
